# Supplementary material for: Calculating the quality of public high-throughput sequencing data to obtain a suitable subset for reanalysis from the Sequence Read Archive
Source: Gigascience. 2017 Apr 25;6(6):1–8. doi: 10.1093/gigascience/gix029 (PMC5459929; doi:10.1093/gigascience/gix029)
Supplement: GIGA-D-17-00014_Original_Submission.pdf [file gix029_GIGA-D-17-00014_Original_Submission.pdf]

Manuscript

Click here to download Manuscript ohta_gigascience_manuscript.pdf

Calculating quality of public high-throughput sequencing data to obtain suitable subset for reanalysis from the Sequence Read Archive

Tazro Ohta[1], Takeru Nakazato[1], and Hidemasa Bono[1]

[1]Database Center for Life Science, Joint Support-Center for Data Science research, Research Organization of Information and Systems, Mishima, Shizuoka 411-8540, Japan

Correspondence should be addressed to T.O. (t.ohta@dbcls.rois.ac.jp) and H.B. (bono@dbcls.rois.ac.jp).

Abstract

Background: It is important for public data repositories to promote the reuse of archived data. In the growing field of omics science, however, the increasing number of submissions of high-throughput sequencing (HTSeq) data to public repositories prevents users from choosing a suitable data set from among the large number of search results. Repository users need to be able to set a threshold to reduce the number of results to obtain a suitable subset of high-quality data for reanalysis.

Results: We calculated the quality of sequencing data archived in a public data repository, the Sequence Read Archive (SRA), by using the quality control

software FastQC. We obtained quality values for 1,171,313 experiments, which can be used to evaluate the suitability of data for reuse. We also visualized the data distribution in SRA by integrating the quality information and metadata of experiments and samples.

Conclusions: We provide quality information of all of the archived sequencing data which enable users to obtain sufficient quality sequencing data for reanalyses. The calculated quality data are available to the public in various formats. Our data also provide an example of enhancing the reuse of public data by adding metadata to published research data by a third party.

Keywords

high-throughput sequencing, sequencing quality, public data, database

Background

The publication of primary data used as evidence is essential for ensuring transparency and reproducibility in scientific research, but also important for promoting the reuse of data in future research activities [1,2]. In the last decade, the rapid advance of high-throughput DNA sequencing (HTSeq) technologies has enabled omics research projects to produce massive amounts of data, which have huge potential for reuse from different perspectives [3]. An increasing number of sets of omics data are being produced by not only international consortiums, but also individual research projects [4]. However, only a portion

of all archived data derived from large projects are frequently being reused, in contrast to data from individual studies. This is because users prefer to collect data from a single project that had a sufficient number of samples and that were produced by experiments under reliable conditions, thus ensuring the quality of the data. To promote the reuse of combined sets of data from multiple projects, public repositories have to provide a filtering feature in data searches, so that users can control the number of experiments and quality of the data in their searches. Currently, data searches provided by repositories based on metadata described by the data submitter cannot be used for filtering by data quality. To enable such filtering, repositories have to provide information on the quality of sequence data.

As the number of submissions of data to repositories increases, the number of search results produced by inputting the same query also increases. To select an appropriate amount of data, sequencing quality is usually used to ensure that the data are sufficient for an analysis; however, there are currently no detailed quality information available for public data. For example, when a user searches with the query “transcriptome data of mouse brain” against the Sequence Read Archive (SRA), a public HTSeq data repository, over 120,000 experiments are shown in the search results. To reduce the number of results and thus obtain the most suitable data set for analysis, the user needs to download all of the data and calculate the sequence quality, for instance, the read length or number of

reads. Given the rapid increase in the amount of archived data, this is becoming increasingly unfeasible.

Providing information on data quality can also provide an insight into the data repository itself. Basic quality values, for example, mean and median levels of sequencing throughput, read length, or base call accuracy of the specific sequencing method, are important to obtain an overview of the archive. These values can be used to illustrate the overall distribution of data in the repository. The distribution can show the standard of data quality; thus, a user can use these values to filter out inappropriate data sets from among the thousands of search results.

Here, we provide the calculated sequencing quality data of all archived HTSeq experiments to allow repository users to control the amount and the quality of data in their searches. We also performed analyses to visualize the distribution of archived data by quality values, to show the standard of data quality in the repository.

Data description

Downloading of sequencing data

To calculate quality values of sequencing data, we downloaded the data from the SRA, which is the largest public repository for HTSeq data [5]. Sequencing data containing personally identifiable information that should be shared in a controlled-access manner are not archived in SRA. In this study, we

downloaded open-access SRA data stored in FASTQ format from the FTP server of the DNA Data Bank of Japan.

We analyzed all of the publicly available HTSeq data submitted to SRA up until December 2015. The total number of sequenced samples was 1,171,313 and the number of sequenced bases was more than 2.7 trillion. The varieties of sequencing methods, sequencing instruments, and sequenced sample organisms are shown in Figure 1, which were extracted from the metadata described by the data submitter. The most common sequencing method is the whole-genome shotgun (WGS) approach, which was employed for 426,841 samples, or 36.4% of the total. The number of different sequenced organisms is 33,961, based on the Taxonomy ID. The most commonly sequenced organism in SRA is human, with 216,896 samples, or 18.5% of the total, while the total number of samples whose scientific name contains “metagenome” is 244,457, or 20.9% of the total. The number of experiments counted by the sequencing instrument model used shows that Illumina HiSeq 2000 is the most commonly used instrument in SRA, with 542,332 experiments, or 46.3% of the total.

Calculation of sequence read quality

To enable filtering of the search results in the repository by quality information, we extracted sequence read quality values from raw sequencing data using FastQC. FastQC is one of the most popular software programs for performing quality control of high-throughput sequencing data [6]. By using the results

from FastQC, we calculated comparable values of sequence data, such as the total number of reads, mean and median sequence read length, %GC, read duplicate percentage, mean and median base call accuracy, and percentage of failed base calling (N content) (Table 1). The read quality values were calculated for each downloaded set of sequencing run data in FASTQ format, and then assembled using the SRA Experiment ID.

We integrated the categorical values described in metadata of the sample and experiment with calculated read quality data. Experimental metadata were extracted from an SRA metadata XML file downloaded from the FTP server of the National Center for Biotechnology Information (NCBI). Sample information was extracted from the XML file downloaded from BioSample, a database maintained by the International Nucleotide Sequence Database Collaboration (INSDC) to archive information on biological materials [7].

Analyses

The state of the HTSeq repository visualized by the distribution of data quality

Providing sequence data quality enables users to control the number of search results from a data repository. The integration of information on data quality with metadata of samples and experiments can be used to develop a better search function. However, to offer a method of obtaining a suitable data set from thousands of search results, it is necessary to know the standard of data

quality and the data distribution in the repository. To illustrate the state of publicly available HTSeq data using quality values, histograms were created for sequencing throughput, base call accuracy, and N content (Fig. 2, Supplementary Fig. 1). As Fig. 1 shows, there is a huge bias in numbers of sequencing methods, sequenced organisms, and used sequencing instruments. Thus, we focused on the factor that defines the range of the quality values, not the count of data, which is probably affected by the bias of the number of sequencing instruments. To understand the data attribute that is decisive to its distribution, histograms were color-coded in terms of the metadata of sequencing experiments and sequenced sample organisms. In the histograms of sequencing throughput, library source, particularly genomic, transcriptomic, or metagenomic source of sequencing, clearly explains the distribution of sequenced bases (Fig. 2a, 2b, Supplementary Fig. 2). Overall, the mean value of throughput was $2.371e^{+09}$ and the median value was $3.349e^{+08}$. In the histogram of base call accuracy, as expected, the values are strongly affected by the choice of sequencing chemistry (Fig. 2c, 2d, Supplementary Fig. 3). The mean value of base call accuracy was 29.45, while the median value was 35.52. The histogram drawn by N content showed that 1,103,515 items, namely, 94.2% of the data, had N at less than 1% of the total sequences (Supplementary Fig. 3). For the data with a higher proportion of N content, there may have been an error in the sample DNA preparation or sequencing operation.

Data distribution by read quality for each sequencing method

SRA accepts the submission of various kinds of sequencing data, such as those obtained by WGS, RNA-Seq, ChIP-Seq, and metagenomic approaches, as well as many other DNA library construction strategies. To accomplish higher measurement accuracy and greater dynamic range, each sequencing method has ideal conditions regarding sequencing quality. We analyzed the distribution of data in each data set by a library strategy to investigate how many performed experiments achieved such ideal conditions. We employed 988,678 sets of data for this analysis, which were obtained by the sequencing of human samples via WGS, amplicon sequencing, RNA-Seq, ChIP-Seq, pooled clone sequencing, or whole-exome sequencing (WXS). We visualized the data distribution by creating a histogram for each library strategy (Figure 3). The histograms were color-coded by the sequencing instrument manufacturer to show which type of sequencing chemistry had been selected. In one of the six library strategies, namely, amplicon sequencing, multiple types of sequencing chemistry were used, while the others were performed mostly by the Illumina sequencing chemistry. The histograms indicate that the five library strategies require a larger number of sequence reads and higher base call quality. In contrast, experiments by other library strategies were performed with a short read length of around 100 bases long, while some amplicon sequencing experiments were performed with longer sequence reads of hundreds of bases. A total of 66.3% of amplicon sequencing experiments were performed by non-Illumina sequencers,

for which the average read length was 388.4. This is consistent with the standards of each sequencing strategy [8].

Changes of sequencing quality during SRA's history

Since 2007, when the first next-generation sequencing data were submitted to the SRA, there have been rapid advances in the sequencing technology regarding both the instruments and the chemistry, which have significantly improved the quality of sequencing data. The improved specs of sequencers have enabled various new sequencing methods to be developed, but have also helped improve the data quality output by existing methods. We visualized the changes of quality values for each sequencing method over time. A change in four sequencing qualities, total number of reads, read length, sequencing throughput, and base call quality, of six library strategies, WGS, amplicon, RNA-Seq, ChIP-Seq, pooled clone, and WXS, are visualized by box plots in quarterly time series (Fig. 4, Supplementary Fig. 4). While the plots of pooled clone sequencing could not be evaluated due to a lack of continuous data submission, the plots of the other strategies show their trends over time. The plots of amplicon sequencing show no specific tendency, probably indicating that such sequencing quality values are determined by the characteristics of each sequencing project, surveying of which requires more detailed metadata. In ChIP-Seq and WXS, sequencing throughput increased slightly over time. In plots of base call accuracy, ChIP-Seq, RNA-Seq, WGS, and WXS showed

increases of the value, possibly reflecting the improvement of sequencing technologies.

Discussion

The increasing number of submissions of data to public high-throughput sequencing data repositories has made it difficult to reuse the published data efficiently. By calculating quantitative variables of sequencing data and integrating them with information on experiments and sample organisms, we enabled an appropriate size of subset to be obtained from multiple projects archived in the repository. Without any quantitative information, users cannot choose a reliable data set from among thousands of search hits. When users search data with a query of sample-related information, such as a treatment of biological materials, the number of search results tends to be very small or too large for users to be able to browse through, due to the lack of detailed metadata. In contrast, our results can provide information in a way that enables users to look into a large data set and control the amount of data output by their search by setting a threshold regarding the quality value.

Our approach also enables visualization of the data distribution to find the relative position of data in a data set of similar features. Moreover, it is now possible to show the distribution of read information and its change over time for each sequencing method. These features are useful when deciding on the conditions to set in a sequencing experiment. For example, from the results of

our investigation on the distribution of sequencing throughput and base call accuracy, users can check whether the total number of sequenced bases is within the appropriate range for one's library source, and can also evaluate whether the base call accuracy is sufficient to follow the standard quality of the instruments used.

The data of FastQC that we used to calculate the read information are also published on our web server (http://sra.dbcls.jp/fastqc). These data enable SRA users to examine read quality information before downloading sequencing data from the FTP server. They can also help users to avoid downloading data that do not match their objectives, which can decrease the cost of downloading. The quantitative metadata produced and summarized in this study are also published as linked open data, which can be accessed via the SPARQL endpoint, to allow users to integrate these with other public biological data resources related to the SRA.

Our study shows that efforts to extend metadata of existing public sequencing data by a third party can increase data accessibility and enhance the reuse of published data. Although it is important to publish primary data used in research, it is not possible to maintain a large repository of high-throughput sequencing data without sufficient economic and human resources [14]. To tackle the problem of the sustainability of data repositories, approaches to decrease the cost of hosting them have been proposed, including a new data compression strategy. As another method of increasing their efficiency, we also highlight the

importance of biological data repositories increasing their value by enhancing the reuse of data. We strongly believe that the use of open data is the best way of keeping them accessible.

Potential implications

The amount and the accuracy of sequencing data is drastically changing in these years. This means that database users have to care about the details of the experiment, for example, date of sequencing or used sequencing equipments for each database entry. The quality information of public sequencing data provided by our work is able to be used to evaluate the reliability of entries in biological databases, such as genome variations or gene expressions.

Methods

Data retrieval from the data repository

We downloaded data from the FTP server of the DNA Databank of Japan (ftp.ddbj.nig.ac.jp/ddbj_database/dra) by using the lftp command. Most of the data were downloaded as a FASTQ format file. When data were only available in SRA format, we decompressed the data to FASTQ format by using the fastq-dump command of the SRA toolkit (ver. 2.5.1). fastq-dump is performed with the --split-3 option to split paired-end files into individual FASTQ files. Downloaded data were analyzed by md5 checksum to confirm that they were not corrupt.

Extraction of sequencing quality information

First, we performed FastQC [11] via the command line with options --no-extraction and --threads 4. The versions of FastQC software used in this study were 0.10.0, 0.10.1, and 0.11.3, depending on the date when each sequencing run was performed. We confirmed that there were no differences in the results of the modules that we used among the versions. We parsed the result files of FastQC (fastqc_data.txt) by the bioruby [15] module bio-fastqc [16], which we developed based on biogem [17]. The results from paired-end reads were concatenated by calculating the average values for each quality value, excluding values of the total number of sequences that were summed. If an experiment involved multiple sequencing runs, quality values were also concatenated to create comparable values for each experiment. By using relation of SRA ExperimentID and BioSampleID, calculated quality values, experimental metadata, and sample organism metadata were assembled. The code is available online [18].

Publishing quality data as linked open data

We published the individual results of FastQC for each sequencing run on our web server [19]. Each set of sequencing quality data was converted into RDF format and deposited in the NBDC RDF portal [20]. We developed an ontology

to describe sequencing quality information, namely, sequence statistics ontology, and also published it in the NBDC RDF portal.

Visualization of the data distribution in the repository

Visualization of the distribution of data was performed using R language (ver. 3.2.3) [21] and library ggplot2 (ver. 2.1.0) [22]. The code is available online [18].

Availability of source code and requirements

Project name: SRA Quanto

Project home page: https://github.com/inutano/sra-quanto

Operating systems: Platform independent

Programming language: Ruby, R

Other requirements: Univa Grid Engine 8.4.0 or higher

License: MIT

Any restrictions to use by non-academics: N/A

Availability of supporting data and materials

The dataset supporting the conclusions of this article is available in figshare (https://dx.doi.org/10.6084/m9.figshare.4498907.v1).

Declarations

List of abbreviations

HTSeq: high-throughput sequencing

INSDC: International Nucleotide Sequence Database Collaboration

JST: Japan Science and Technology Agency

NBDC: National Bioscience Database Center

NCBI: National Center for Biotechnology Information

SRA: Sequence Read Archive

WGS: whole-genome shotgun

WXS: whole-exome sequencing

Competing interests

The authors declare that they have no competing interests.

Funding

This work has been supported by the National Bioscience Database Center (NBDC) of the Japan Science and Technology Agency (JST).

Authors’ contributions

Conceptualization: TO TN HB. Methodology, Software, Investigation, Visualization, Writing original draft: TO. Supervision: TN HB.

Acknowledgements

The authors are grateful to Prof. Toshihisa Takagi for helpful comments.

References

1. Organisation for Economic Co-operation and Development. OECD Principles and Guidelines for Access to Research Data from Public Funding. OECD; Paris. 2007. http://www.oecd.org/science/sci-tech/38500813.pdf. Accessed 11 Nov 2016.
2. Sansone SA, Rocca-Serra P, Field D, Maguire E, Taylor C, Hofmann O, Fang H, Neumann S, Tong W, Amaral-Zettler L, Begley K. Toward interoperable bioscience data. Nature genetics. 2012 Feb 1;44(2):121-6
3. Ball CA, Sherlock G, Brazma A. Funding high-throughput data sharing. Nature biotechnology. 2004 Sep 1;22(9):1179-83.
4. Nakazato T, Ohta T, Bono H. Experimental design-based functional mining and characterization of high-throughput sequencing data in the sequence read archive. PLoS One. 2013 Oct 22;8(10):e77910.
5. Kodama Y, Shumway M, Leinonen R. The Sequence Read Archive: explosive growth of sequencing data. Nucleic acids research. 2012 Jan 1;40(D1):D54-6.
6. Simon Andrews. A quality control tool for high throughput sequence data. 2010. http://www.bioinformatics.babraham.ac.uk/projects/fastqc/. Accessed 11 Nov 2016.
7. Barrett T, Clark K, Gevorgyan R, Gorelenkov V, Gribov E, Karsch-Mizrachi I, Kimelman M, Pruitt KD, Resenchuk S, Tatusova T, Yaschenko E. BioProject and BioSample databases at NCBI: facilitating

capture and organization of metadata. Nucleic acids research. 2012 Jan 1;40(D1):D57-63.

8. Goodwin S, McPherson JD, McCombie WR. Coming of age: ten years of next-generation sequencing technologies. Nature Reviews Genetics. 2016 Jun 1;17(6):333-51.
9. Cochrane G, Cook CE, Birney E. The future of DNA sequence archiving. GigaScience. 2012 Jul 12;1(1):1.
10. Goto N, Prins P, Nakao M, Bonnal R, Aerts J, Katayama T. BioRuby: bioinformatics software for the Ruby programming language. Bioinformatics. 2010 Oct 15;26(20):2617-9.
11. Tazro Inutano Ohta, ruby parser for FastQC, a quality control software for high-throughput sequencing data. https://rubygems.org/gems/bio-fastqc Accessed 11 Nov 2016.
12. Bonnal RJ, Aerts J, Githinji G, Goto N, MacLean D, Miller CA, Mishima H, Pagani M, Ramirez-Gonzalez R, Smant G, Strozzi F. Biogem: an effective tool-based approach for scaling up open source software development in bioinformatics. Bioinformatics. 2012 Apr 1;28(7):1035-7.
13. Tazro Inutano Ohta, Summary of quantitative sequence information of the Sequence Read Archive. https://github.com/inutano/sra-quanto. Accessed 14 Nov 2016.
14. Database Center for Life Science. DBCLS SRA. http://sra.dbcls.jp/. Accessed 14 Nov 2016.

15. National Bioscience Database Center. NBDC RDF Portal. https://integbio.jp/rdf/. Accessed 14 Nov 2016.

16. R Core Team. R: A language and environment for statistical computing. R Foundation for Statistical Computing, Vienna, Austria. 2015. https://www.R-project.org/. Accessed 11 Nov 2016.

17. H. Wickham. ggplot2: Elegant Graphics for Data Analysis. Springer-Verlag New York; 2009.

Table

Table 1. Calculated sequence quality values and used modules of FastQC

**Calculated quality value**	**Numbers of multiple runs in an experiment**	**Used FastQC modules**
Total Number of Reads	Added	Basic Statistics module
Mean/Median Read Length	Average	Sequence Length Distribution module
%GC	Average	Basic Statistics module
Total Duplicate Percentage	Average	Duplicate Sequences module
Mean/Median Base Call Accuracy	Average	Per Base Sequence Quality module
N Content	Average	Per Base N Content module

Figure legends

Figure 1. Performed sequencing experiments and sequenced samples of public data for quality calculation. (a) Bar plot of the top 20 library strategies. Values are categorical, retrieved from metadata described by the data submitter. (b) Bar plot of the top 20 sequenced sample organisms. Taxonomy information

is retrieved from the NCBI taxonomy database and declared by the data submitter. (c) Bar plot of sequencing instrument models.

Figure 2. Data distribution in a public data repository by sequencing quality. (a, b) Histogram of sequencing throughput (a), and one color-coded by library source (b). (c, d) Histogram of base call accuracy (c), and one color-coded by instrument manufacturer (d).

Figure 3. Human data distribution for each library strategy. (a–d) Histograms separated by the top 8 library strategies, color-coded by instrument. Data distribution is by the total number of sequences (a), median read length (b), sequencing throughput (c), and median base call accuracy (d) per experiment.

Figure 4. Change of data distribution by sequencing quality over time. (a, b) Box plots separated by the top 6 library strategies, showing quarterly change. Data distribution is by the sequencing throughput (a) and median base call accuracy (b) per experiment. The numbers in plots indicate the numbers of samples in a row. The lines connecting boxes indicate changes of mean value.

Supplementary Figure 1. Data distribution of sequencing throughput for each set of metadata. (a–e) Histograms of sequencing throughput (a), color-coded by library strategy (b), library source (c), top 20 taxonomic scientific names (d), and instrument manufacturer (e).

Supplementary Figure 2. Data distribution of base call accuracy for each set of metadata. (a–e) Histograms of base call accuracy (a), color-coded by library strategy (b), library source (c), top 20 taxonomic scientific names (d), and instrument manufacturer (e).

Supplementary Figure 3. Data distribution by N content. (a–f) Histograms of N content percentage per experiment. Histograms of base call failure without color-coding (a), color-coded by library strategy (b), library source (c), sample organism (d), instrument manufacturer (e), and year of data submission (f). Bins with more than 10,000 counts were removed.

Supplementary Figure 4. Change of data distribution by sequencing quality over time. Box plot of sequence quality per experiment over time. (a) Data distribution by total number of sequence reads per experiment. (b) Data distribution by median sequence read length per experiment.

Figure 1

Click here to download Figure

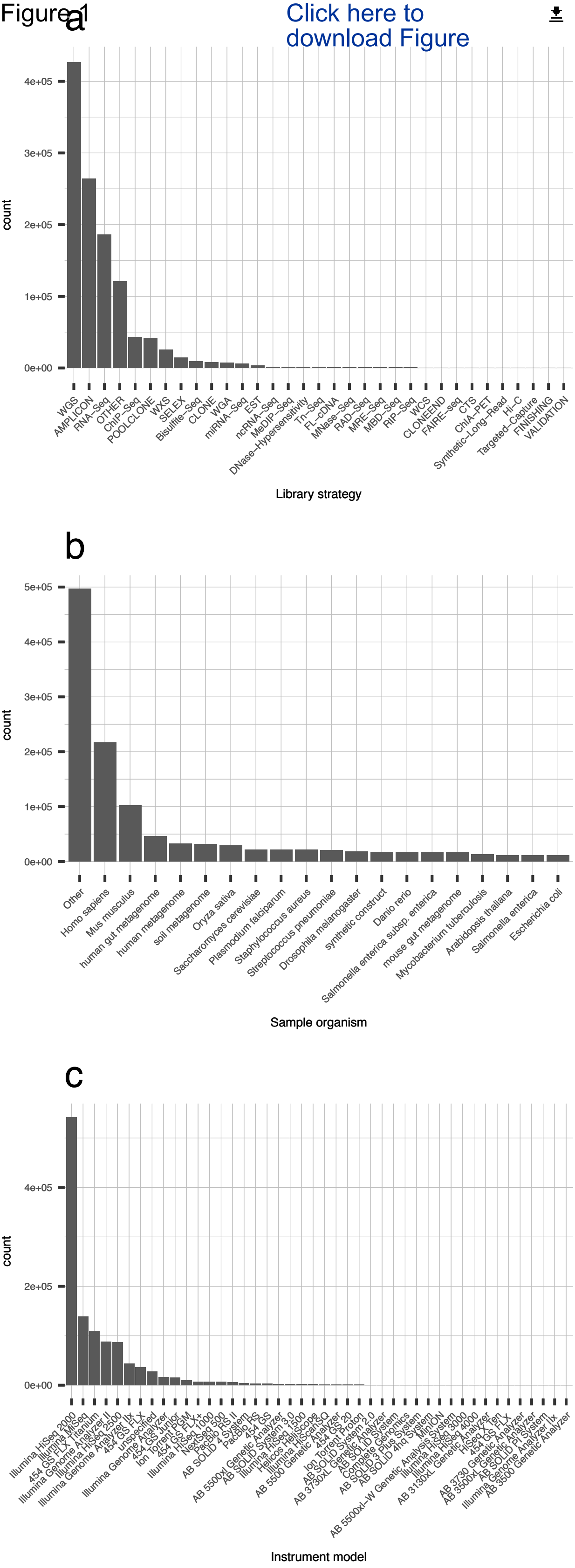

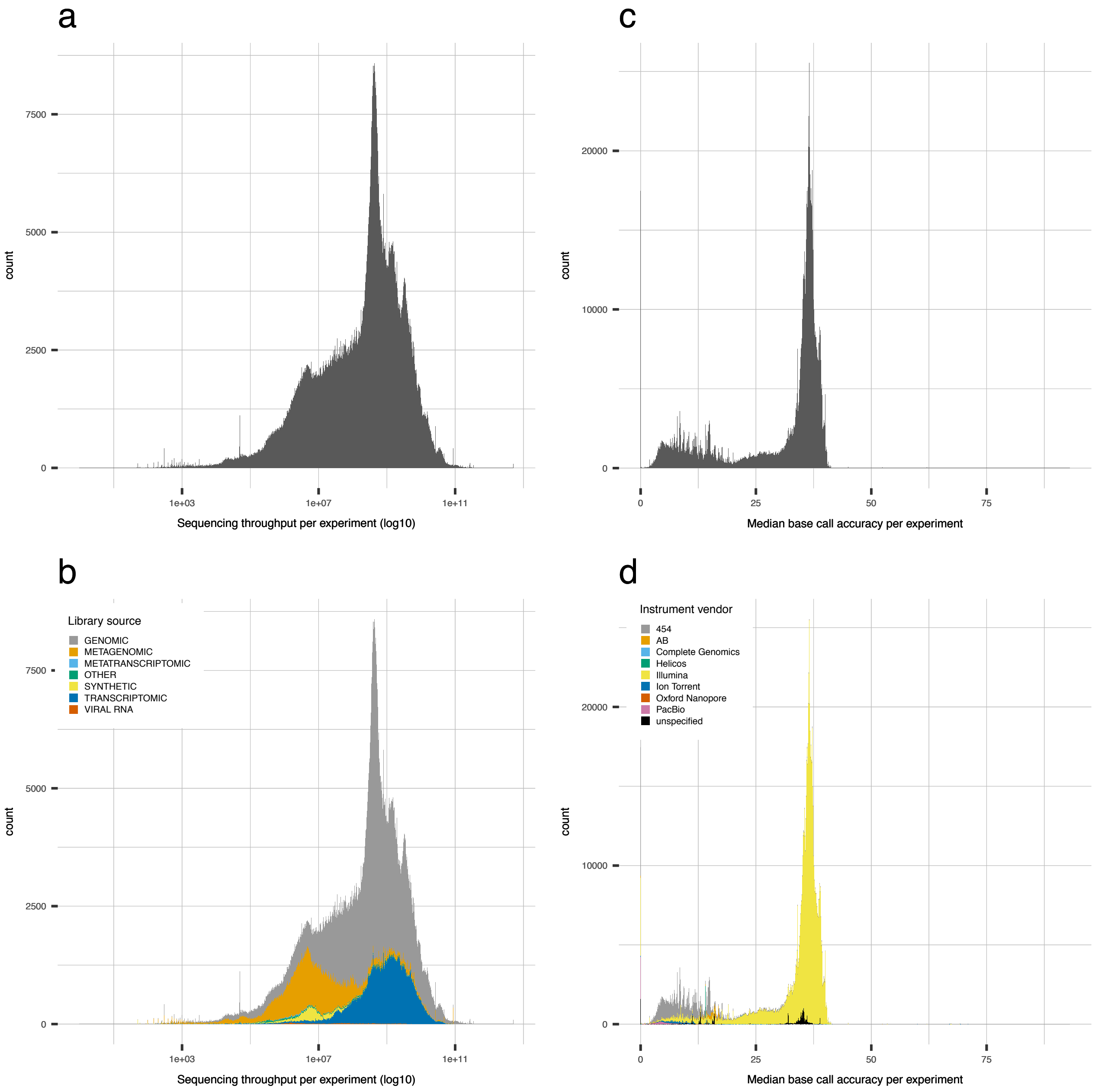
a
7500
5000
2500
0
count
1e+03
1e+07
1e+11
Sequencing throughput per experiment (log10)
c
20000
10000
0
count
0
25
50
75
Median base call accuracy per experiment
b
Library source
GENOMIC
METAGENOMIC
METATRANSCRIPTOMIC
OTHER
SYNTHETIC
TRANSCRIPTOMIC
VIRAL RNA
7500
5000
2500
0
count
1e+03
1e+07
1e+11
Sequencing throughput per experiment (log10)
d
Instrument vendor
454
AB
Complete Genomics
Helicos
Illumina
Ion Torrent
Oxford Nanopore
PacBio
unspecified
20000
10000
0
count
0
25
50
75
Median base call accuracy per experiment

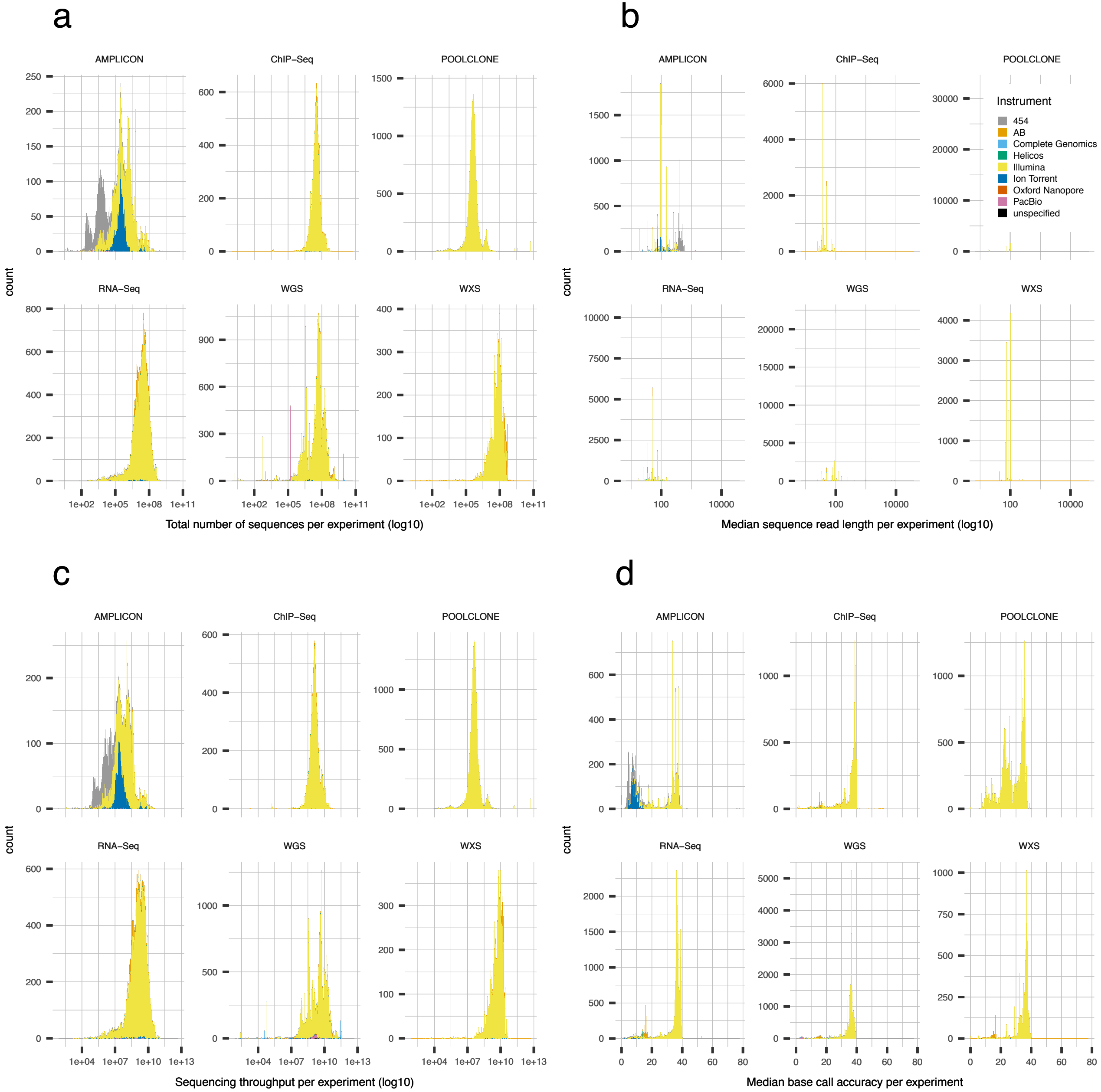
a
b
c
d
AMPLICON
ChIP−Seq
POOLCLONE
RNA−Seq
WGS
WXS
count
Total number of sequences per experiment (log10)
Median sequence read length per experiment (log10)
Sequencing throughput per experiment (log10)
Median base call accuracy per experiment
Instrument
454
AB
Complete Genomics
Helicos
Illumina
Ion Torrent
Oxford Nanopore
PacBio
unspecified

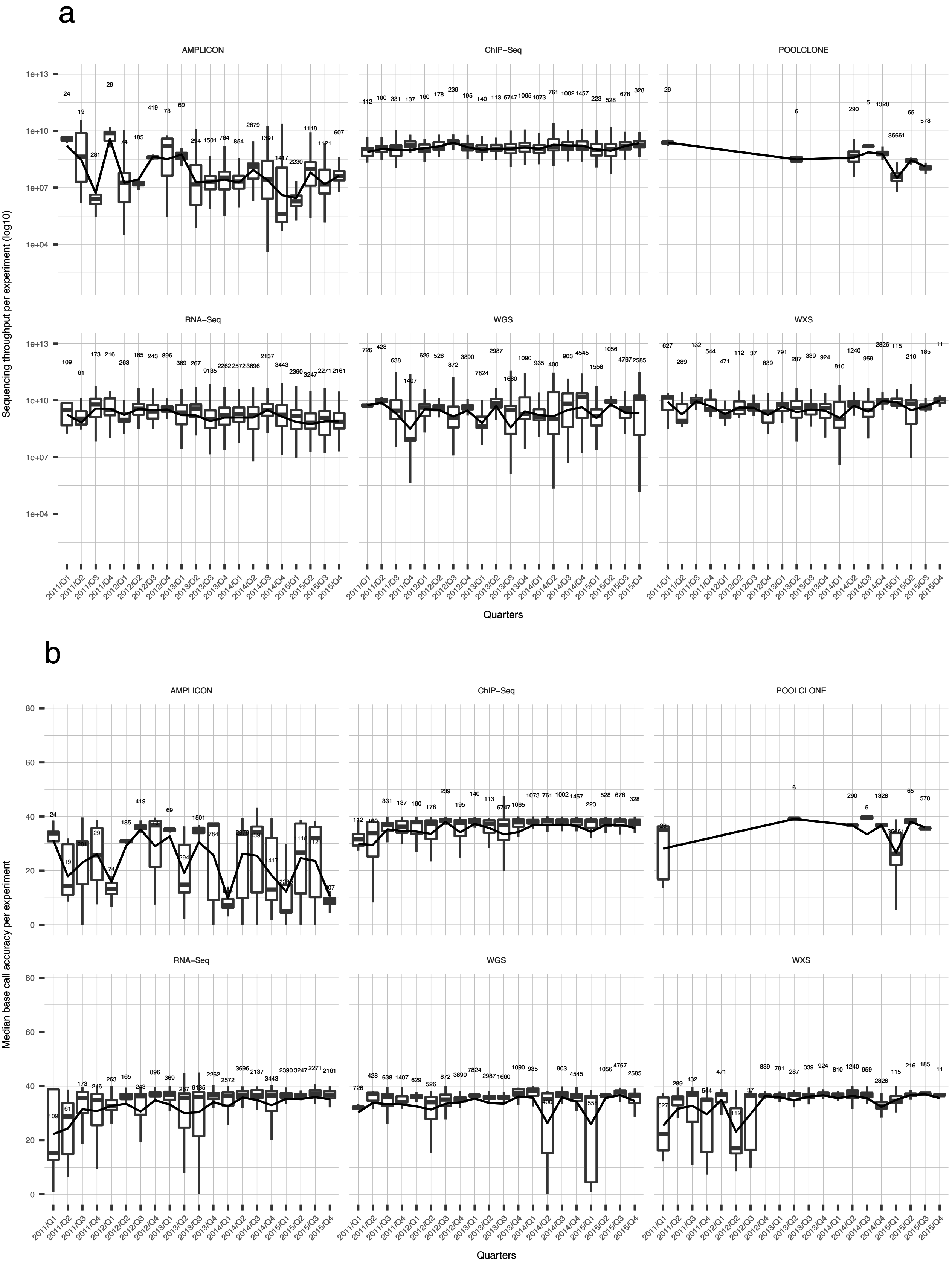
a
AMPLICON
ChIP-Seq
POOLCLONE
RNA-Seq
WGS
WXS
Sequencing throughput per experiment (log10)
1e+13
1e+10
1e+07
1e+04
Quarters
b
Median base call accuracy per experiment
80
60
40
20
0
2011/Q1
2011/Q2
2011/Q3
2011/Q4
2012/Q1
2012/Q2
2012/Q3
2012/Q4
2013/Q1
2013/Q2
2013/Q3
2013/Q4
2014/Q1
2014/Q2
2014/Q3
2014/Q4
2015/Q1
2015/Q2
2015/Q3
2015/Q4

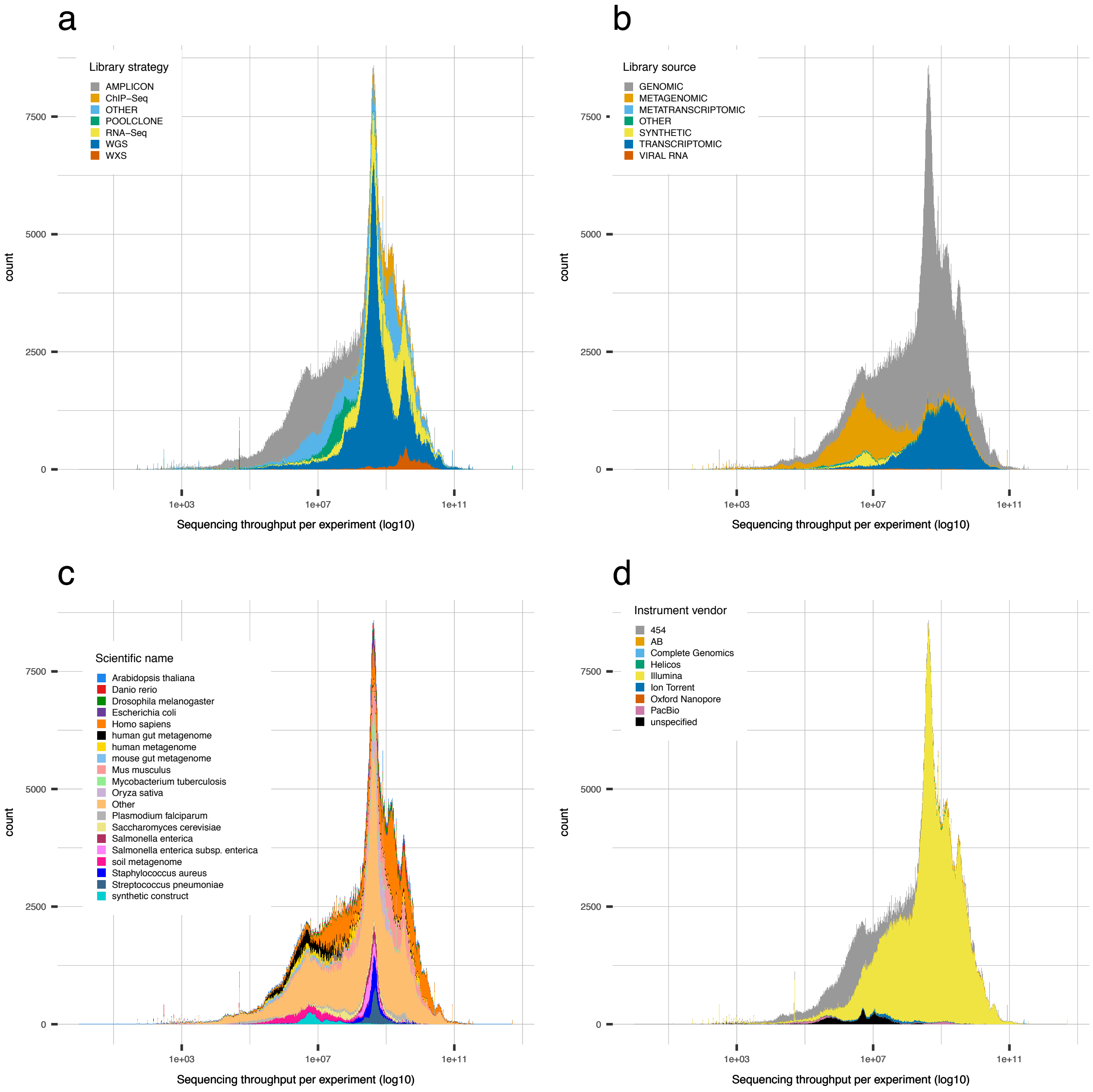
a
Library strategy
AMPLICON
ChIP–Seq
OTHER
POOLCLONE
RNA–Seq
WGS
WXS
count
0
2500
5000
7500
1e+03
1e+07
1e+11
Sequencing throughput per experiment (log10)
b
Library source
GENOMIC
METAGENOMIC
METATRANSCRIPTOMIC
OTHER
SYNTHETIC
TRANSCRIPTOMIC
VIRAL RNA
count
Sequencing throughput per experiment (log10)
c
Scientific name
Arabidopsis thaliana
Danio rerio
Drosophila melanogaster
Escherichia coli
Homo sapiens
human gut metagenome
human metagenome
mouse gut metagenome
Mus musculus
Mycobacterium tuberculosis
Oryza sativa
Other
Plasmodium falciparum
Saccharomyces cerevisiae
Salmonella enterica
Salmonella enterica subsp. enterica
soil metagenome
Staphylococcus aureus
Streptococcus pneumoniae
synthetic construct
count
Sequencing throughput per experiment (log10)
d
Instrument vendor
454
AB
Complete Genomics
Helicos
Illumina
Ion Torrent
Oxford Nanopore
PacBio
unspecified
count
Sequencing throughput per experiment (log10)

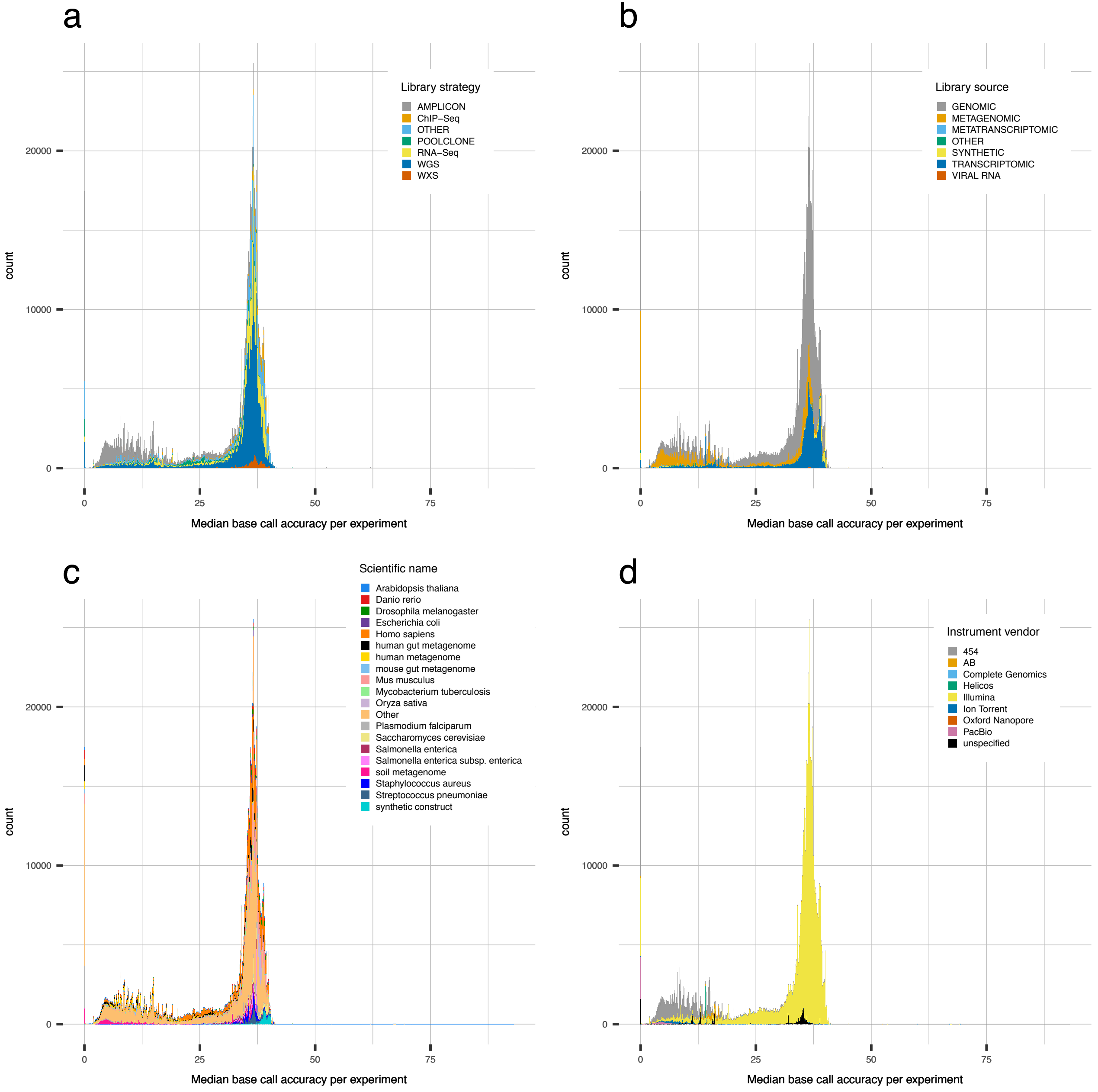
a
Library strategy
AMPLICON
ChIP−Seq
OTHER
POOLCLONE
RNA−Seq
WGS
WXS
count
20000
10000
0
0
25
50
75
Median base call accuracy per experiment
b
Library source
GENOMIC
METAGENOMIC
METATRANSCRIPTOMIC
OTHER
SYNTHETIC
TRANSCRIPTOMIC
VIRAL RNA
count
20000
10000
0
0
25
50
75
Median base call accuracy per experiment
c
Scientific name
Arabidopsis thaliana
Danio rerio
Drosophila melanogaster
Escherichia coli
Homo sapiens
human gut metagenome
human metagenome
mouse gut metagenome
Mus musculus
Mycobacterium tuberculosis
Oryza sativa
Other
Plasmodium falciparum
Saccharomyces cerevisiae
Salmonella enterica
Salmonella enterica subsp. enterica
soil metagenome
Staphylococcus aureus
Streptococcus pneumoniae
synthetic construct
count
20000
10000
0
0
25
50
75
Median base call accuracy per experiment
d
Instrument vendor
454
AB
Complete Genomics
Helicos
Illumina
Ion Torrent
Oxford Nanopore
PacBio
unspecified
count
20000
10000
0
0
25
50
75
Median base call accuracy per experiment

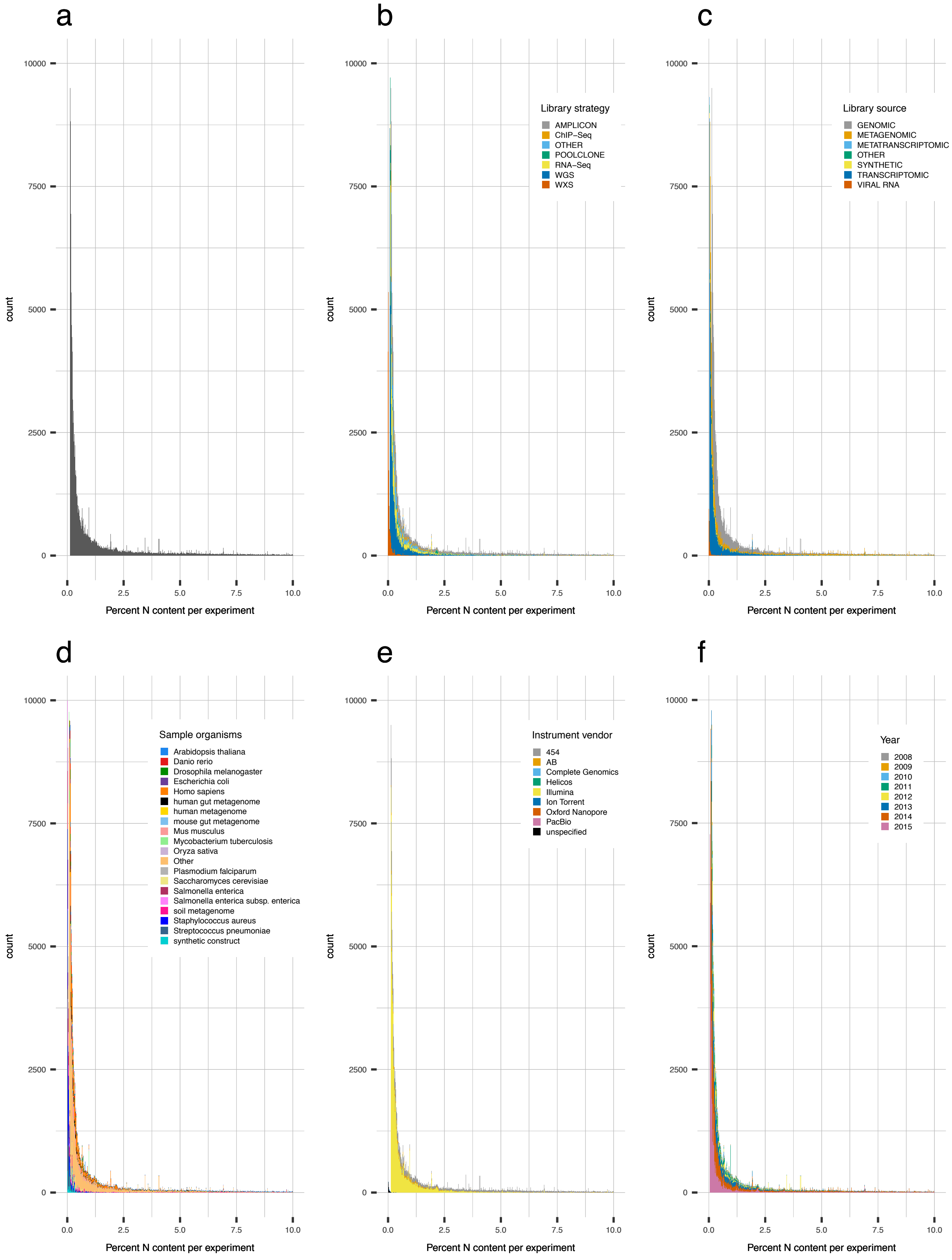

a
b
c
d
e
f
count
0
2500
5000
7500
10000
0.0
2.5
5.0
7.5
10.0
Percent N content per experiment
Library strategy
AMPLICON
ChIP–Seq
OTHER
POOLCLONE
RNA–Seq
WGS
WXS
Library source
GENOMIC
METAGENOMIC
METATRANSCRIPTOMIC
OTHER
SYNTHETIC
TRANSCRIPTOMIC
VIRAL RNA
Sample organisms
Arabidopsis thaliana
Danio rerio
Drosophila melanogaster
Escherichia coli
Homo sapiens
human gut metagenome
human metagenome
mouse gut metagenome
Mus musculus
Mycobacterium tuberculosis
Oryza sativa
Other
Plasmodium falciparum
Saccharomyces cerevisiae
Salmonella enterica
Salmonella enterica subsp. enterica
soil metagenome
Staphylococcus aureus
Streptococcus pneumoniae
synthetic construct
Instrument vendor
454
AB
Complete Genomics
Helicos
Illumina
Ion Torrent
Oxford Nanopore
PacBio
unspecified
Year
2008
2009
2010
2011
2012
2013
2014
2015

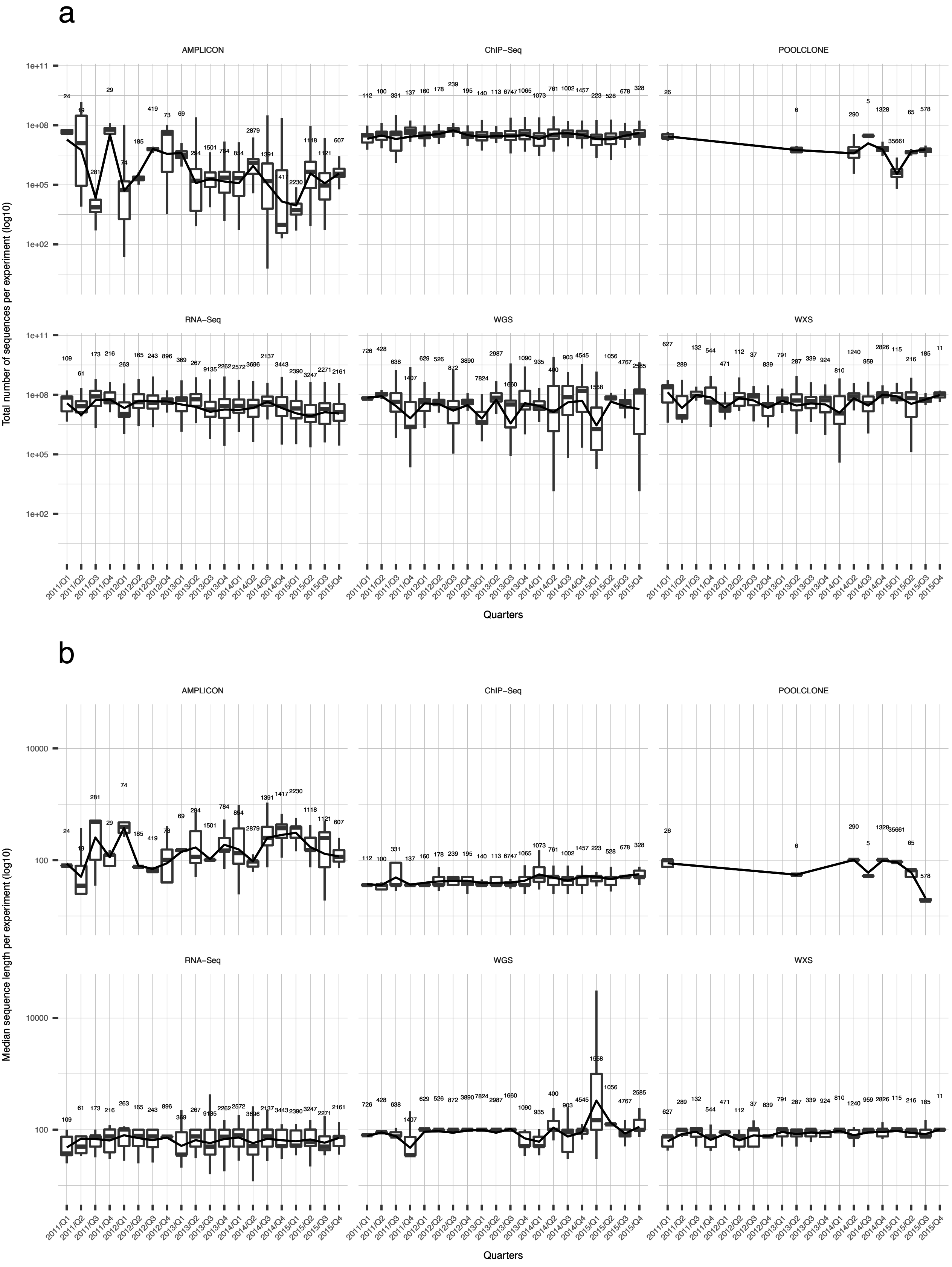

a
AMPLICON
ChIP-Seq
POOLCLONE
RNA-Seq
WGS
WXS
Total number of sequences per experiment (log10)
Quarters
b
AMPLICON
ChIP-Seq
POOLCLONE
RNA-Seq
WGS
WXS
Median sequence length per experiment (log10)
Quarters

Figure 2

Click here to download Figure figure2.pdf

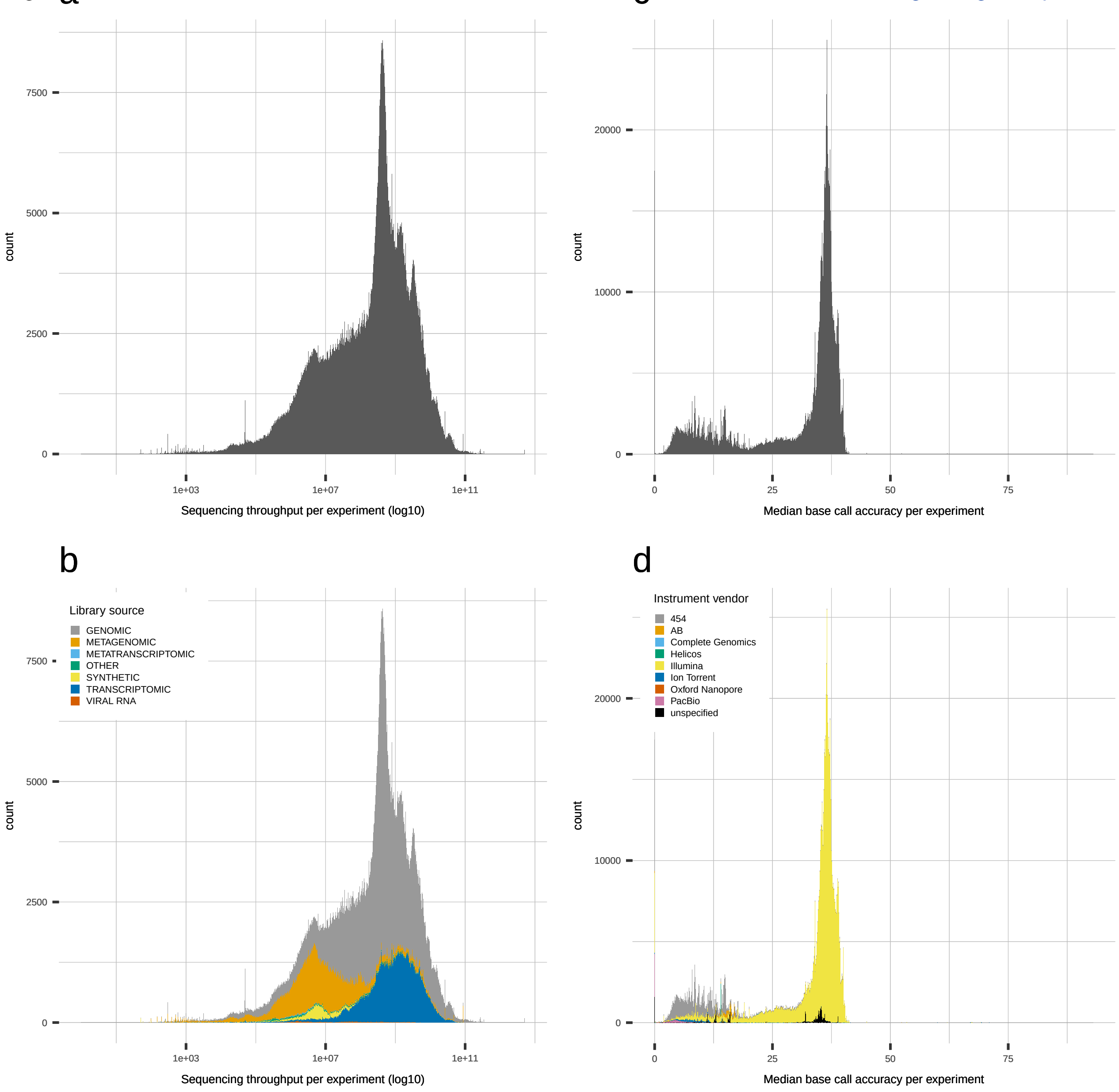

Figure 3

Click here to download Figure figure3.pdf

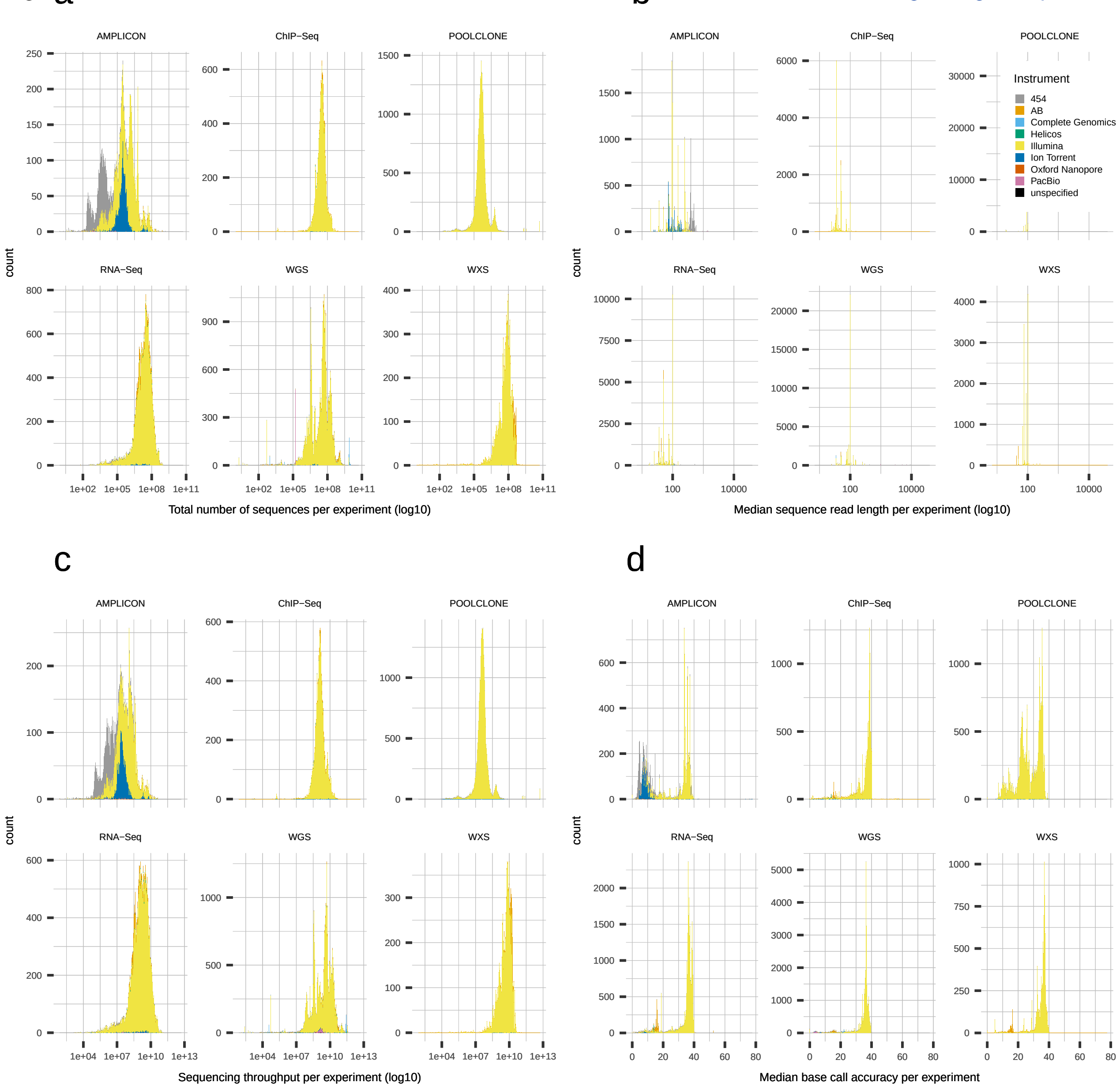

Figure 4 Click here to download Figure figure4.pdf

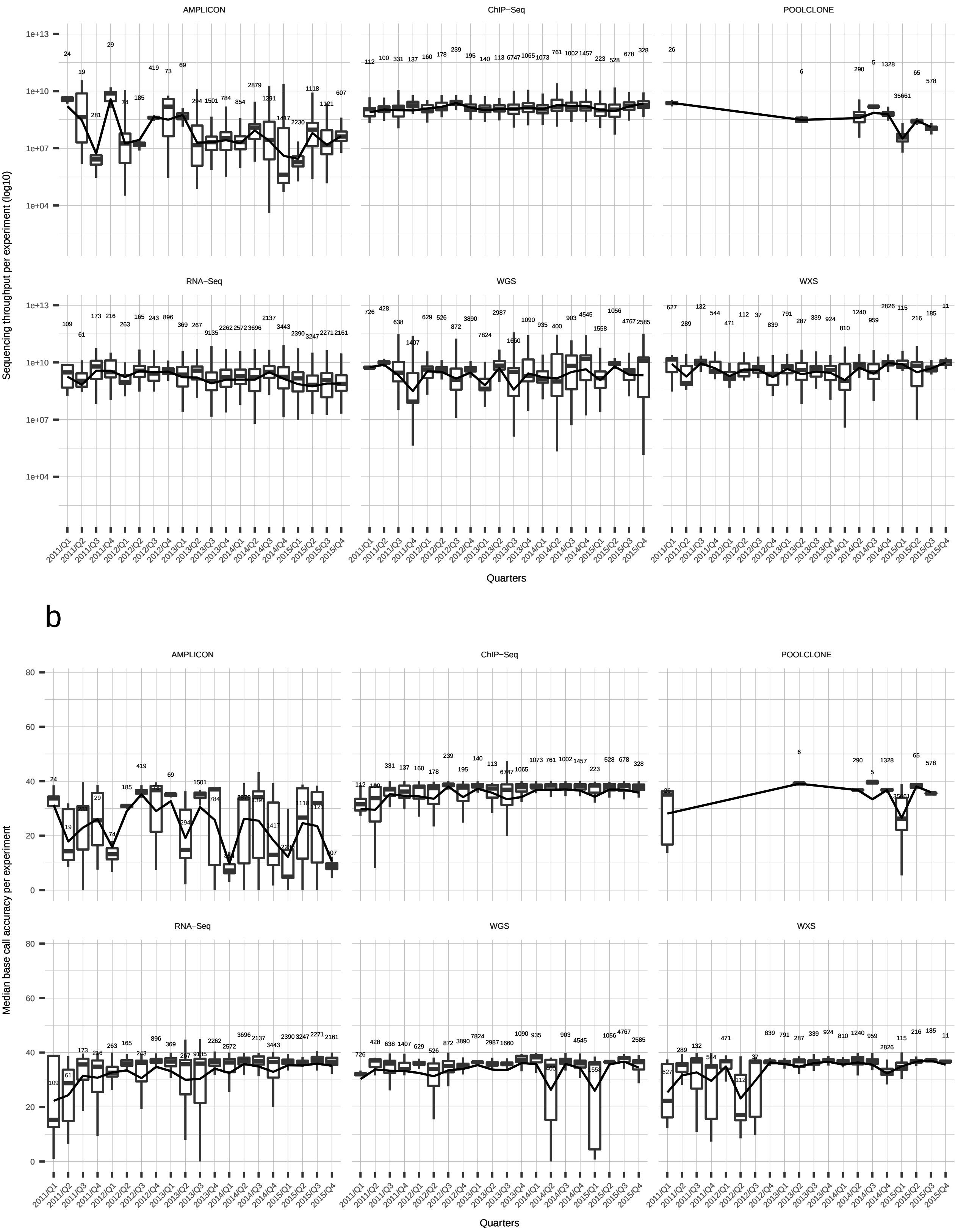

Supplementary figure 1

Click here to access/download
Supplementary Material
supplementary_figure1.pdf

Supplementary figure 2

Click here to access/download
Supplementary Material
supplementary_figure2.pdf

Supplementary figure 3

Click here to access/download
Supplementary Material
supplementary_figure3.pdf

Supplementary figure 4

Click here to access/download
Supplementary Material
supplementary_figure4.pdf

Cover letter
Click here to download Personal Cover ohta_gigascience_coverletter.pdf

DBCLS Database Center for Life Science

Dr. Laurie Goodman, PhD
Editor-in-Chief
GigaScience

January 14, 2017

Dear Dr. Goodman:

We are pleased to submit the attached manuscript entitled: **"Calculating quality of public high-throughput sequencing data to obtain suitable subset for reanalysis from the Sequence Read Archive"** for consideration for publication in *GigaScience* as a research article.

In this manuscript, we propose to researchers to obtain public high-throughput DNA sequencing data for reanalysis from the public data repository, Sequence Read Archive, based on the sequencing quality that we calculated. We also showed the state of the repository by visualizing data statistics based on the type of sequencing methods. Since the result of analysis and our data are encouraging reuse of published data, we believe the manuscript is relevant to the focus of *GigaScience*.

We confirm that this manuscript has not been published elsewhere and is not under consideration by any other journal. All of the authors agree with submission to *GigaScience*. We have no conflicts of interest to declare. We would like to recommend the following reviewers for our manuscript:

Dr. Guy Cochrane, EMBL-EBI, cochrane@ebi.ac.uk
Dr. Simon Andrews, Babraham Institute, simon.andrews@babraham.ac.uk
Dr. Itoshi Nikaido, Riken, itoshi.nikaido@riken.jp

Thank you for your consideration of our manuscript. I look forward to hearing from you.

Sincerely yours,

Tazro Ohta
Project Researcher
Database Center for Life Science (DBCLS)
Research Organization of Information and Systems (ROIS)
t.ohta@dbcls.rois.ac.jp
