# Supplementary figures and images for: Calculating the quality of public high-throughput sequencing data to obtain a suitable subset for reanalysis from the Sequence Read Archive
Source: Gigascience. 2017 Apr 25;6(6):1–8. doi: 10.1093/gigascience/gix029 (PMC5459929; doi:10.1093/gigascience/gix029)

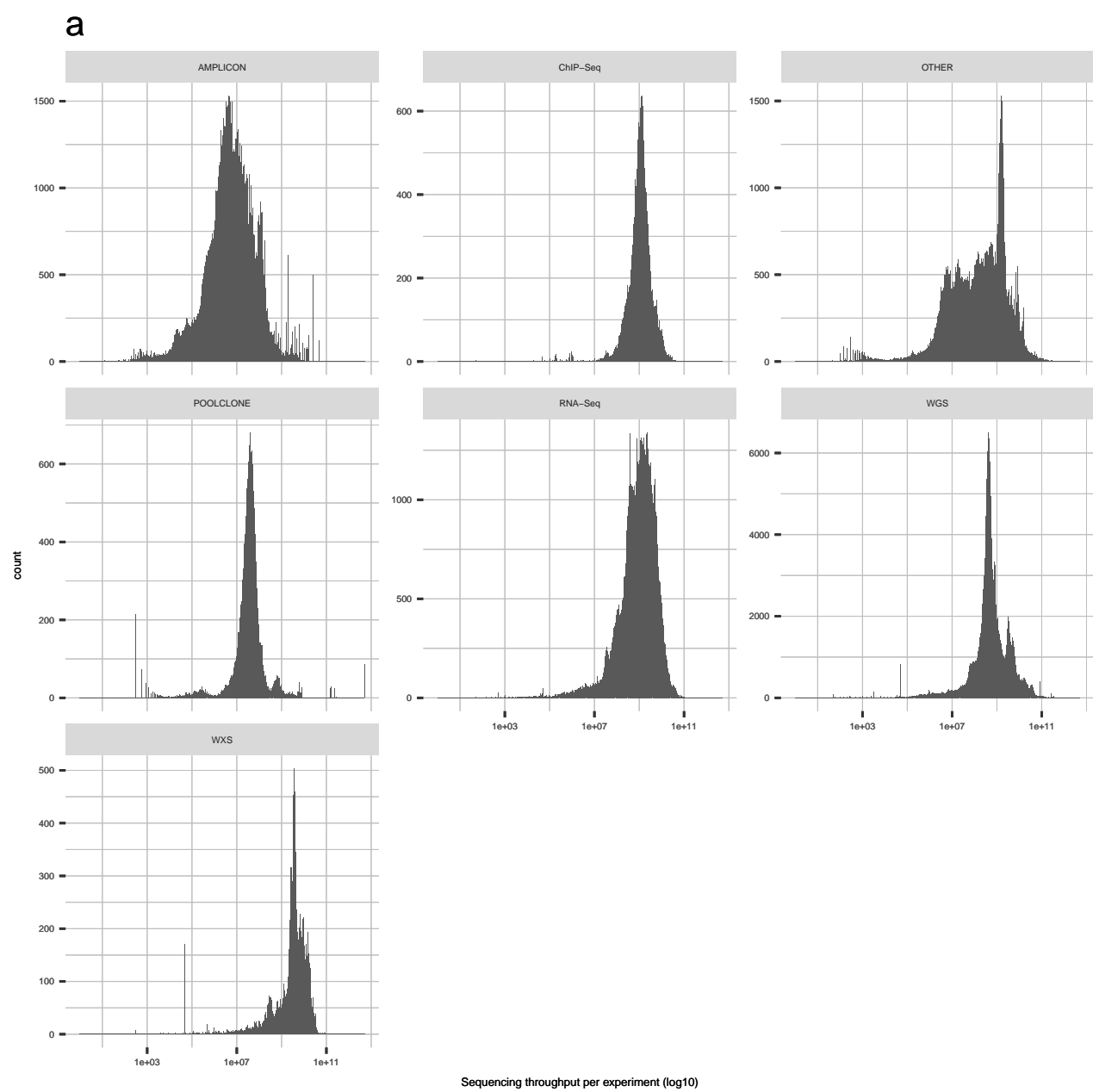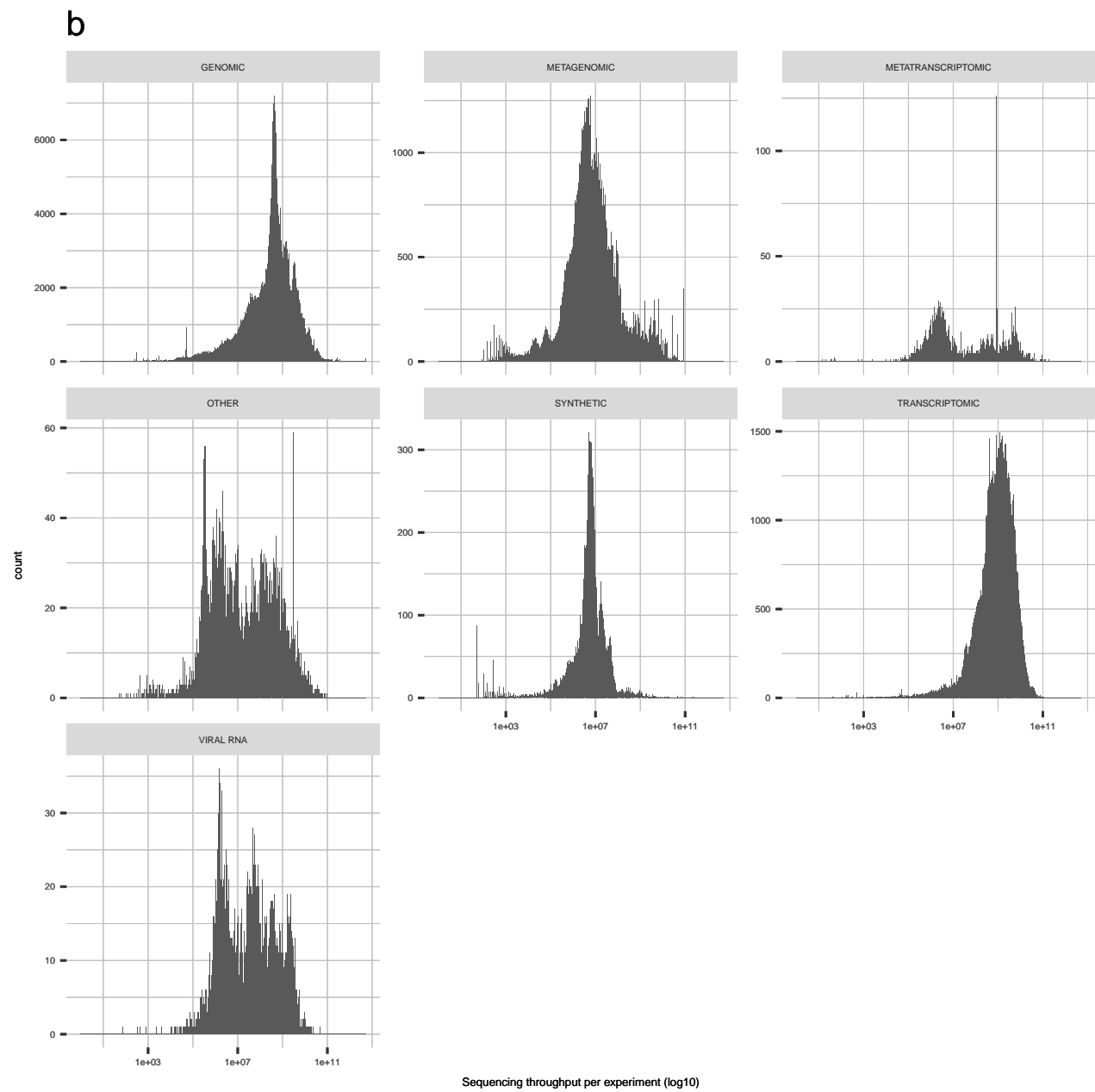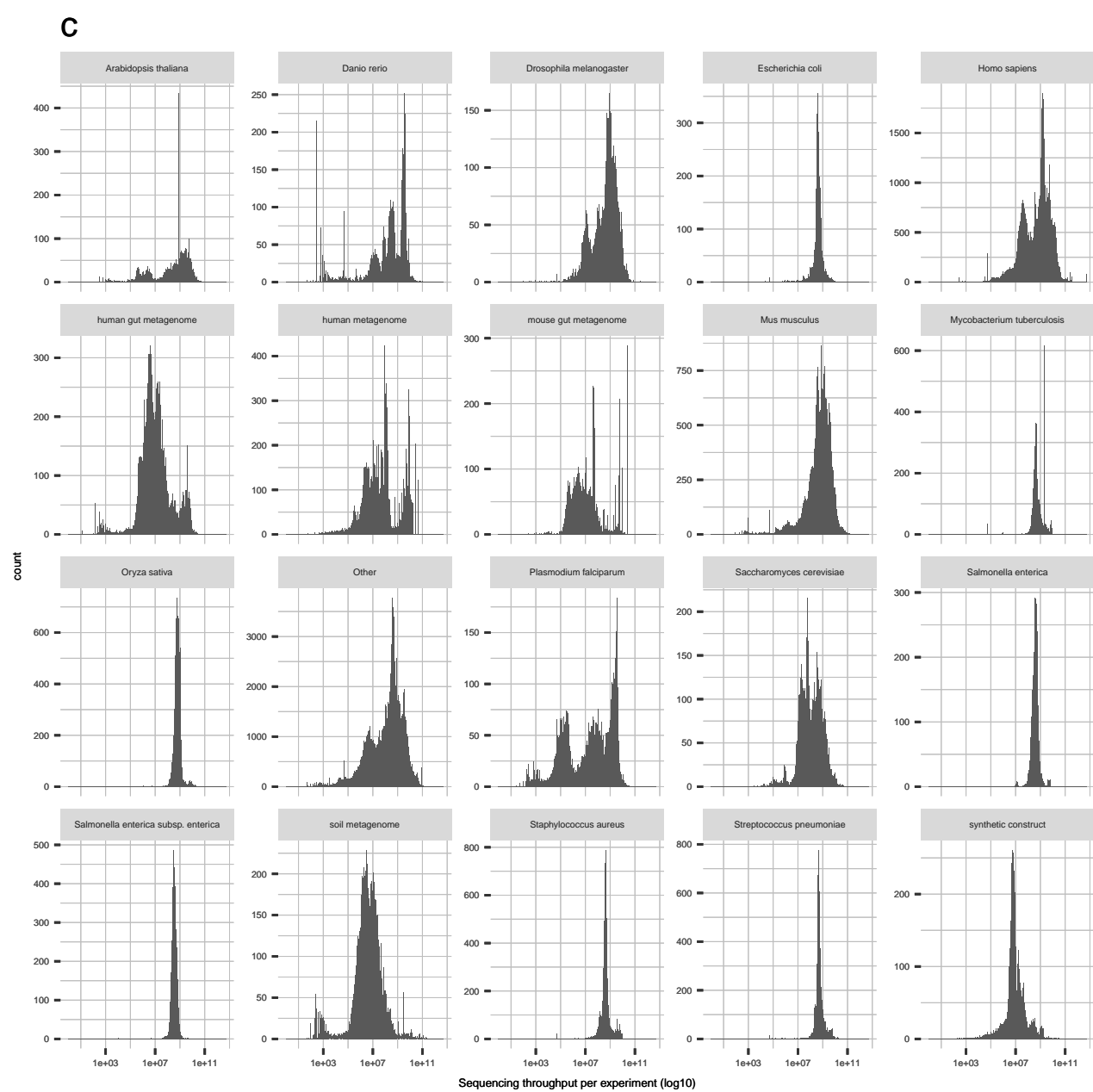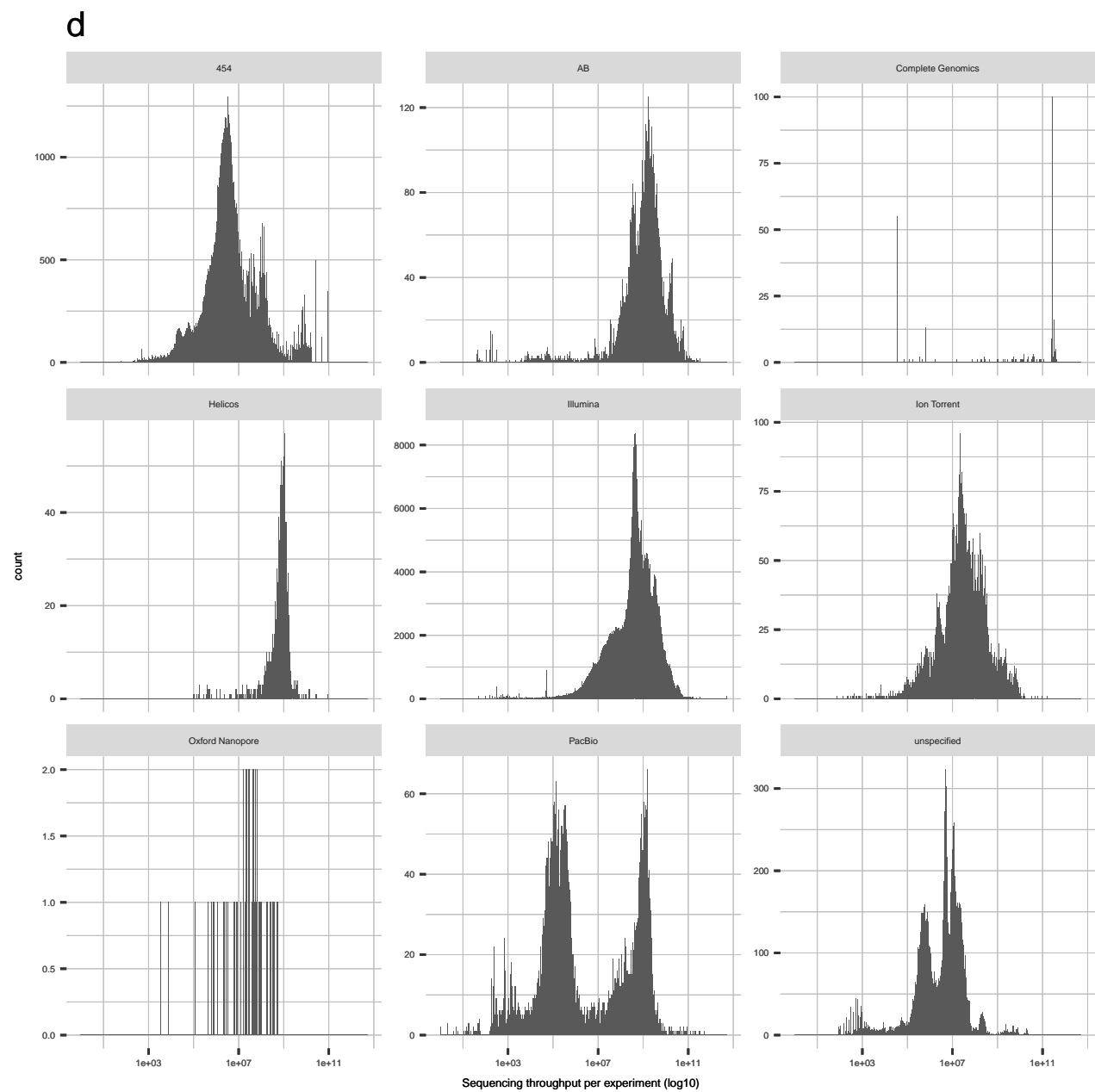

Supplement: Supplemental material — Supplementary Figure 1: Data distribution of sequencing throughput for each set of metadata. (a–e) Histograms of sequencing throughput (a), separated by library strategy (b), library source (c), top 20 taxonomic scientific names (d), and instrument manufacturer (e). Supplementary Figure 2: Data distribution of base call accuracy for each set of metadata. (a–e) Histograms of base call accuracy (a), separated by library strategy (b), library source (c), top 20 taxonomic scientific names (d), and instrument manufacturer (e). Supplementary Figure 3: Data distribution by N content. (a–f) Histograms of N content percentage per experiment. Histograms of base call failure of overall (a), separated by library strategy (b), library source (c), sample organism (d), instrument manufacturer (e), and year of data submission (f). The y-axis is log 10 scale. Supplementary Figure 4: Human data distribution for each library strategy separated by instrument manufacturer. (a–d) Histograms separated by the top 6 library strategies and instrument. Data distribution is by the total number of sequences (a), median read length (b), sequencing throughput (c), and median base call accuracy (d) per experiment. Supplementary Figure 5: Change of data distribution by sequencing quality over time. Box plot of sequence quality per experiment over time. (a) Data distribution by total number of sequence reads per experiment. (b) Data distribution by median sequence read length per experiment. [file gix029_Supp.zip › supplementary_figure1.pdf]

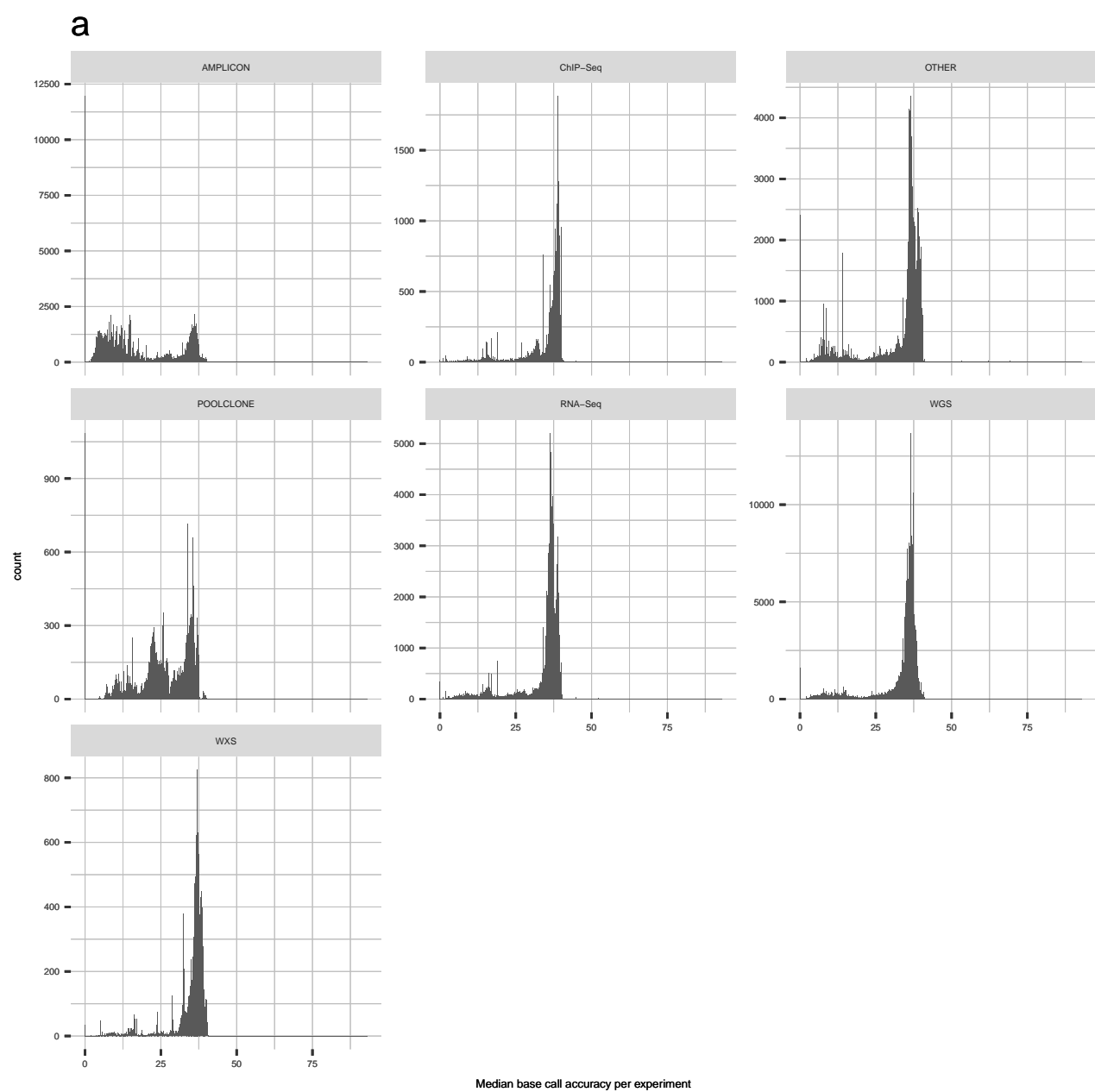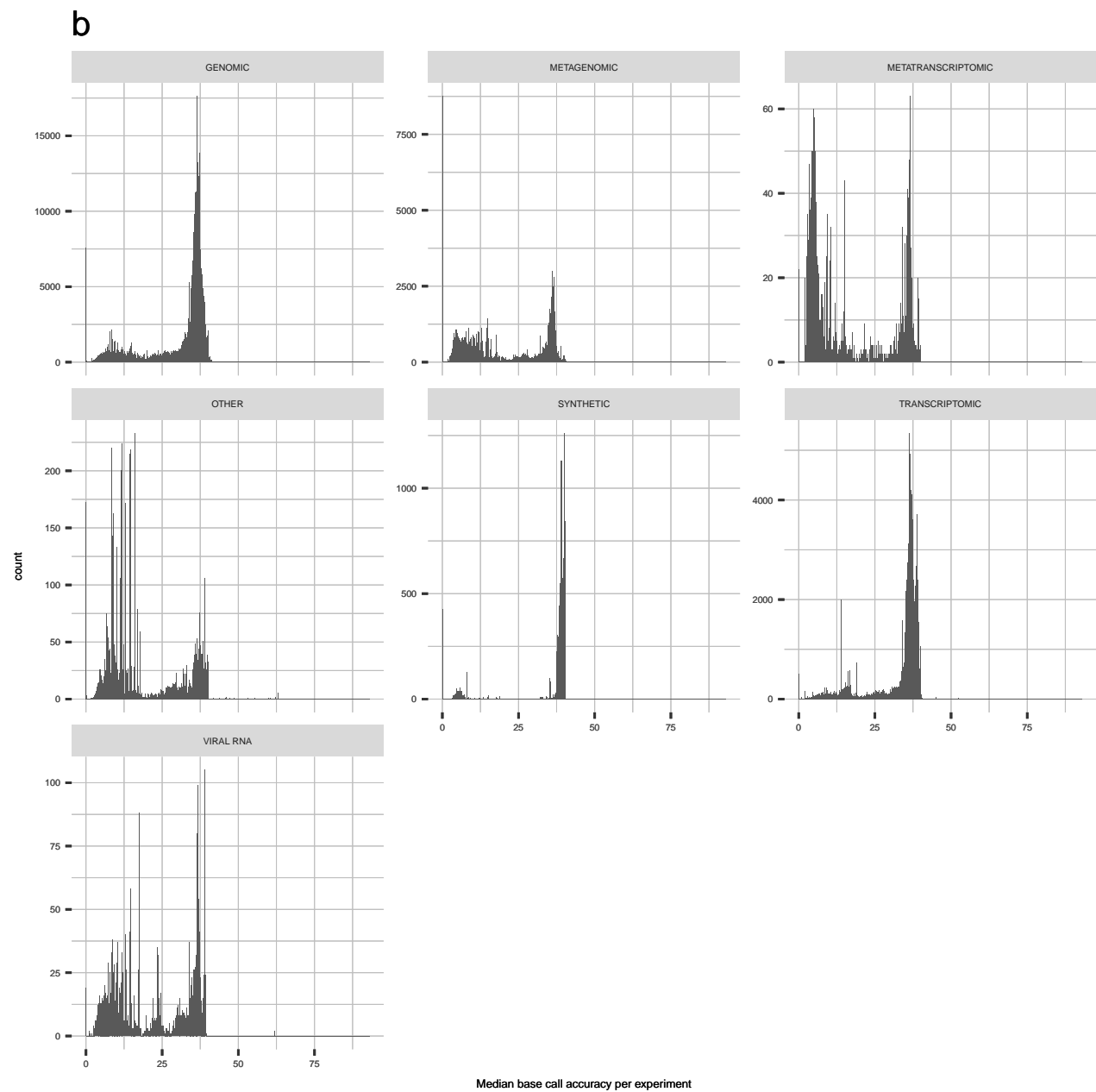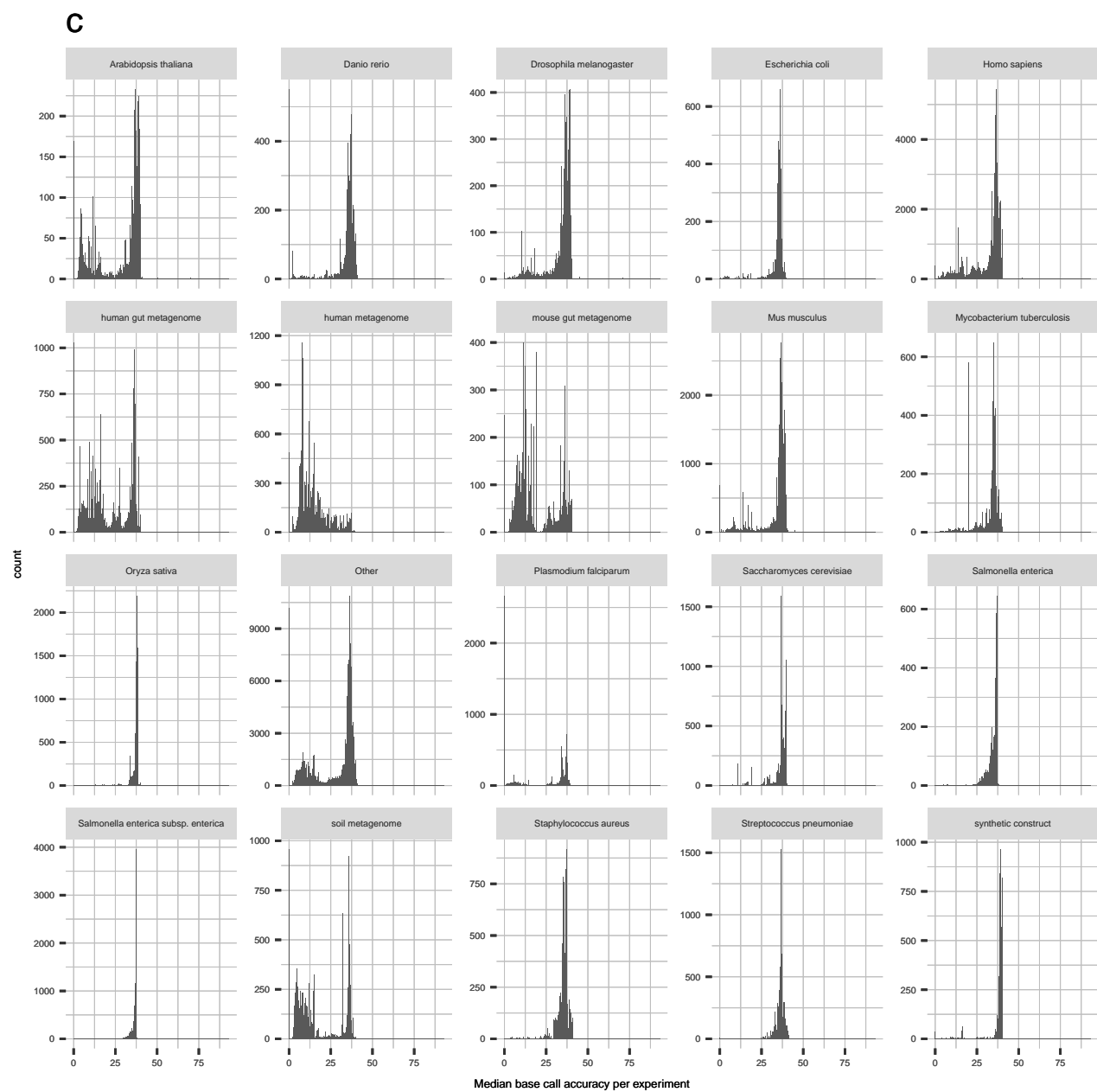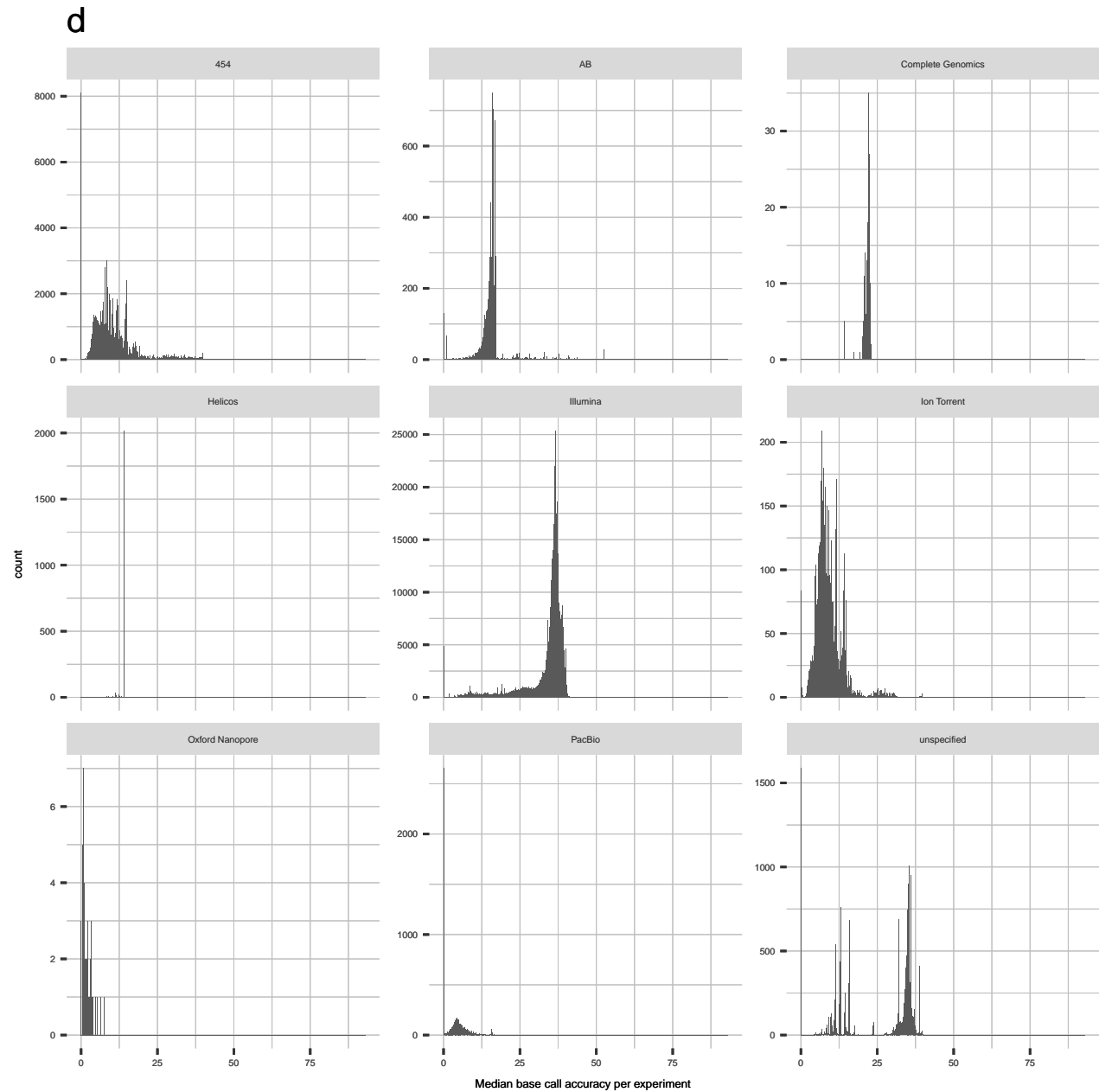

Supplement: Supplemental material — Supplementary Figure 1: Data distribution of sequencing throughput for each set of metadata. (a–e) Histograms of sequencing throughput (a), separated by library strategy (b), library source (c), top 20 taxonomic scientific names (d), and instrument manufacturer (e). Supplementary Figure 2: Data distribution of base call accuracy for each set of metadata. (a–e) Histograms of base call accuracy (a), separated by library strategy (b), library source (c), top 20 taxonomic scientific names (d), and instrument manufacturer (e). Supplementary Figure 3: Data distribution by N content. (a–f) Histograms of N content percentage per experiment. Histograms of base call failure of overall (a), separated by library strategy (b), library source (c), sample organism (d), instrument manufacturer (e), and year of data submission (f). The y-axis is log 10 scale. Supplementary Figure 4: Human data distribution for each library strategy separated by instrument manufacturer. (a–d) Histograms separated by the top 6 library strategies and instrument. Data distribution is by the total number of sequences (a), median read length (b), sequencing throughput (c), and median base call accuracy (d) per experiment. Supplementary Figure 5: Change of data distribution by sequencing quality over time. Box plot of sequence quality per experiment over time. (a) Data distribution by total number of sequence reads per experiment. (b) Data distribution by median sequence read length per experiment. [file gix029_Supp.zip › supplementary_figure2.pdf]

a

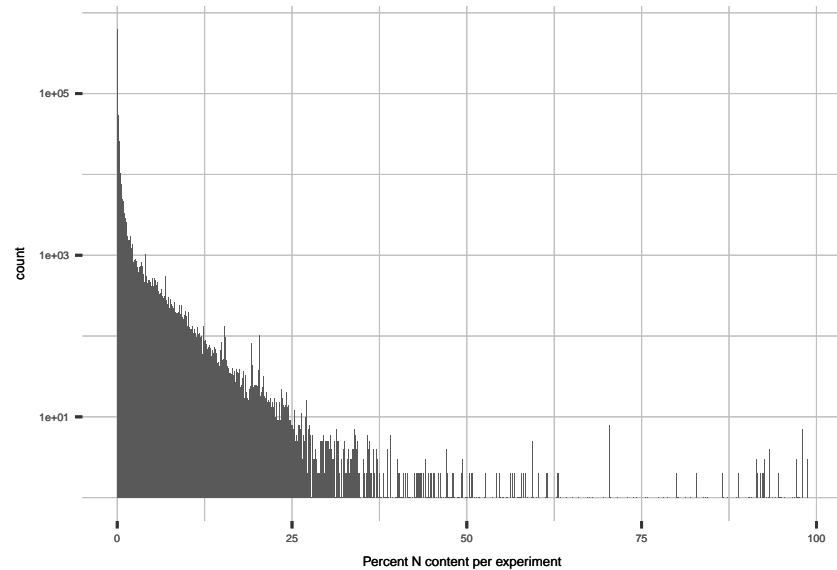

b

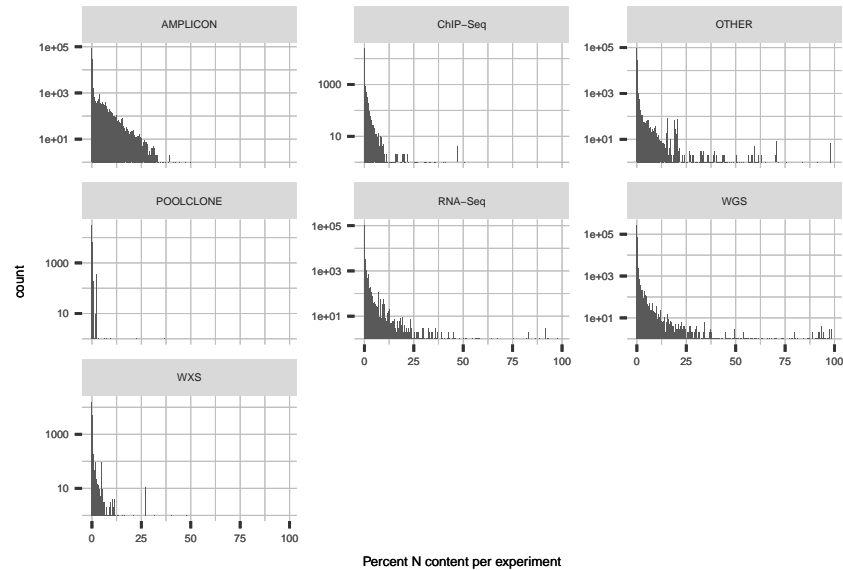

c

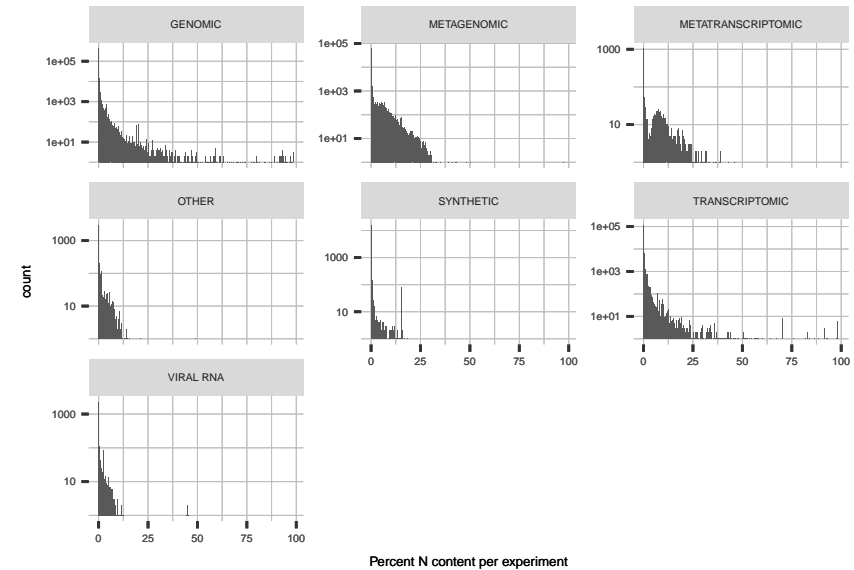

d

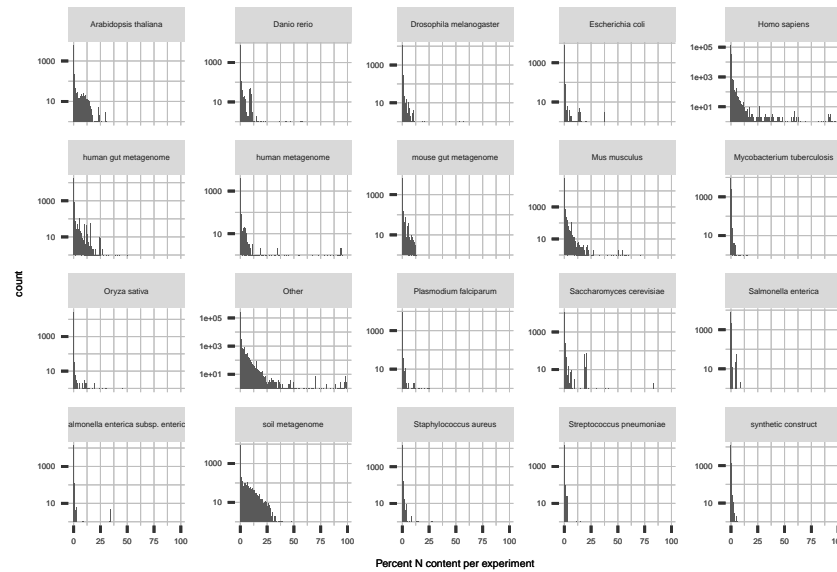

e

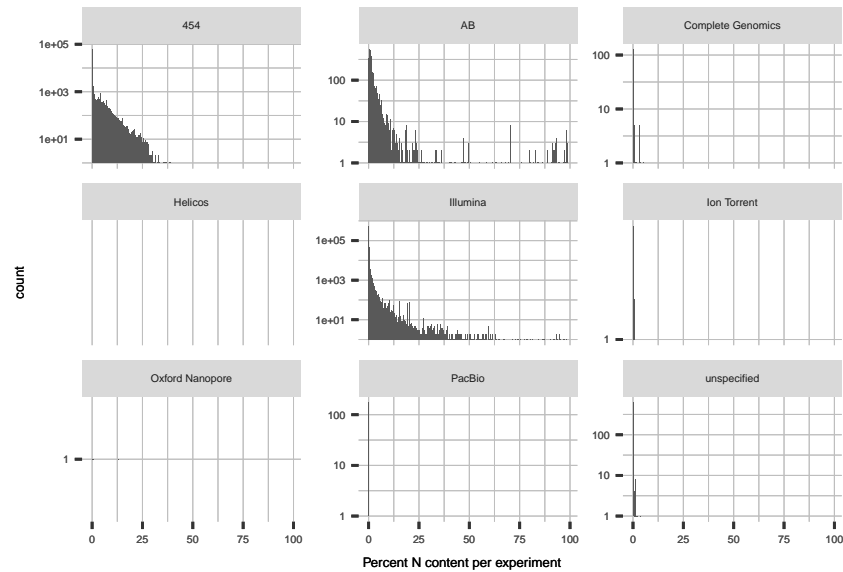

f

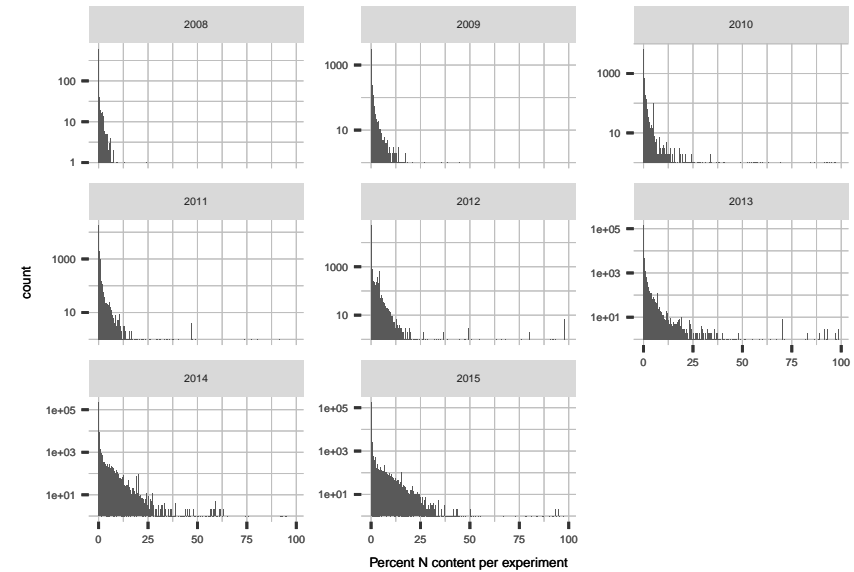

Supplement: Supplemental material — Supplementary Figure 1: Data distribution of sequencing throughput for each set of metadata. (a–e) Histograms of sequencing throughput (a), separated by library strategy (b), library source (c), top 20 taxonomic scientific names (d), and instrument manufacturer (e). Supplementary Figure 2: Data distribution of base call accuracy for each set of metadata. (a–e) Histograms of base call accuracy (a), separated by library strategy (b), library source (c), top 20 taxonomic scientific names (d), and instrument manufacturer (e). Supplementary Figure 3: Data distribution by N content. (a–f) Histograms of N content percentage per experiment. Histograms of base call failure of overall (a), separated by library strategy (b), library source (c), sample organism (d), instrument manufacturer (e), and year of data submission (f). The y-axis is log 10 scale. Supplementary Figure 4: Human data distribution for each library strategy separated by instrument manufacturer. (a–d) Histograms separated by the top 6 library strategies and instrument. Data distribution is by the total number of sequences (a), median read length (b), sequencing throughput (c), and median base call accuracy (d) per experiment. Supplementary Figure 5: Change of data distribution by sequencing quality over time. Box plot of sequence quality per experiment over time. (a) Data distribution by total number of sequence reads per experiment. (b) Data distribution by median sequence read length per experiment. [file gix029_Supp.zip › supplementary_figure3.pdf]

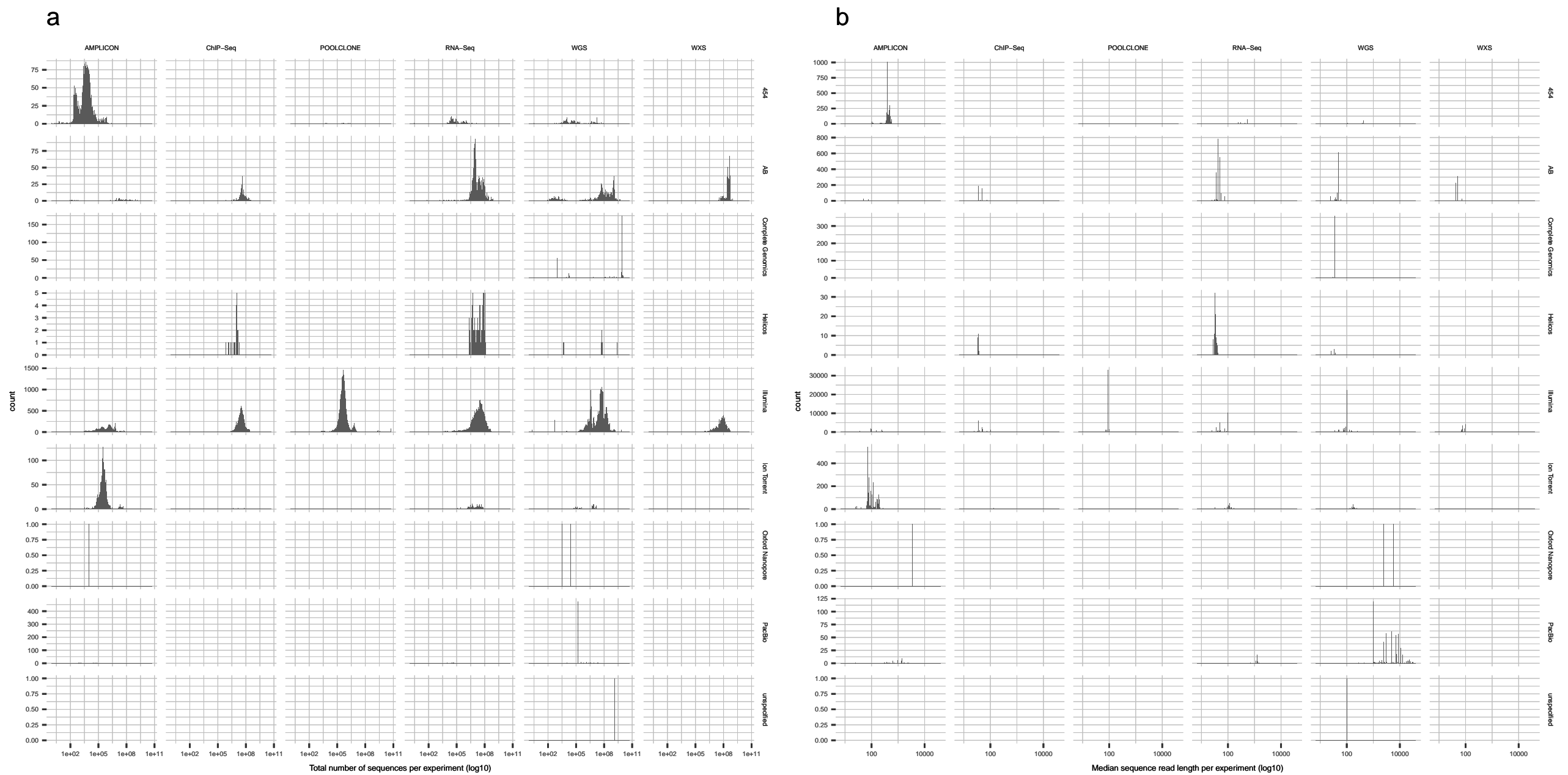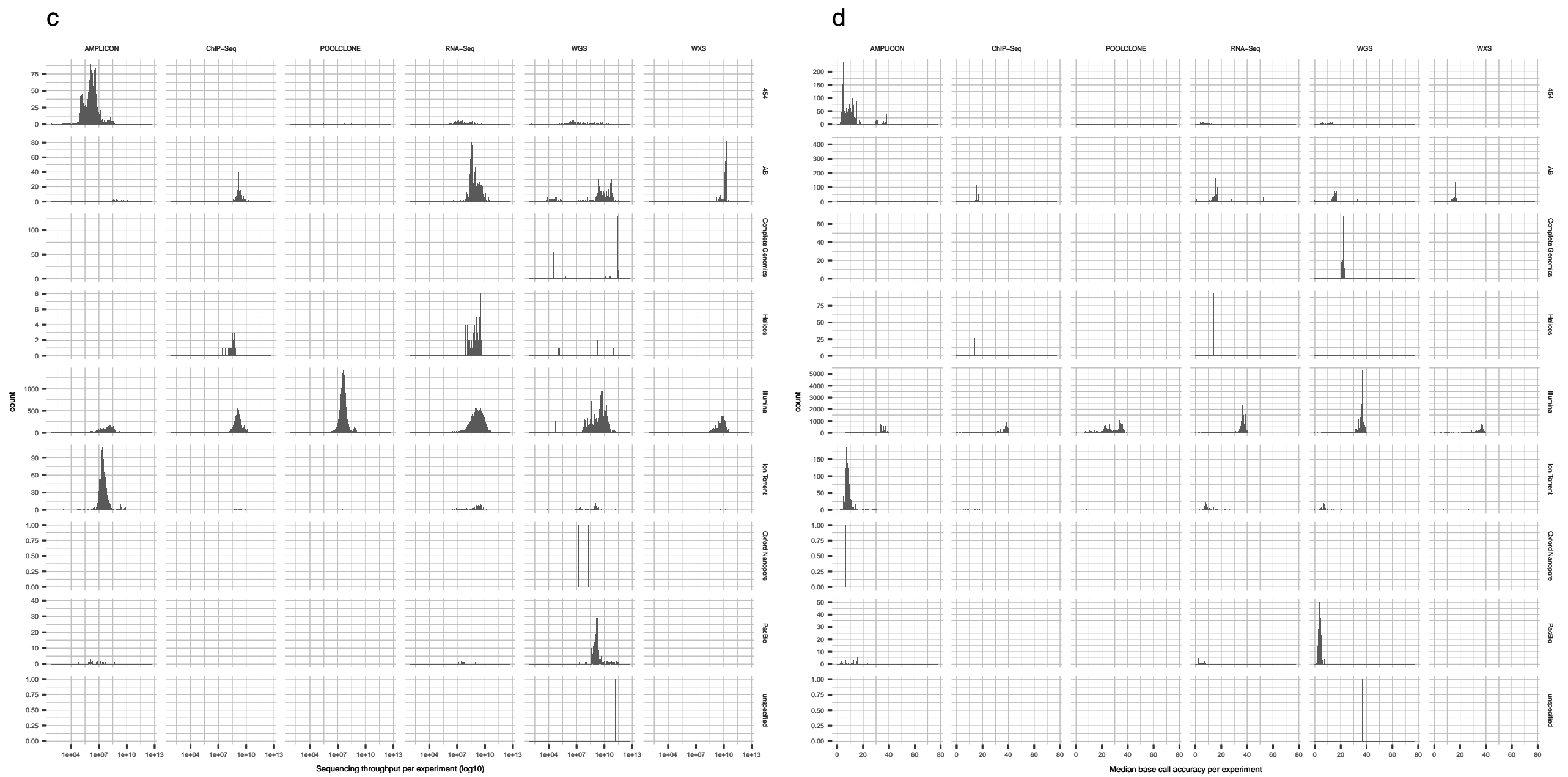

Supplement: Supplemental material — Supplementary Figure 1: Data distribution of sequencing throughput for each set of metadata. (a–e) Histograms of sequencing throughput (a), separated by library strategy (b), library source (c), top 20 taxonomic scientific names (d), and instrument manufacturer (e). Supplementary Figure 2: Data distribution of base call accuracy for each set of metadata. (a–e) Histograms of base call accuracy (a), separated by library strategy (b), library source (c), top 20 taxonomic scientific names (d), and instrument manufacturer (e). Supplementary Figure 3: Data distribution by N content. (a–f) Histograms of N content percentage per experiment. Histograms of base call failure of overall (a), separated by library strategy (b), library source (c), sample organism (d), instrument manufacturer (e), and year of data submission (f). The y-axis is log 10 scale. Supplementary Figure 4: Human data distribution for each library strategy separated by instrument manufacturer. (a–d) Histograms separated by the top 6 library strategies and instrument. Data distribution is by the total number of sequences (a), median read length (b), sequencing throughput (c), and median base call accuracy (d) per experiment. Supplementary Figure 5: Change of data distribution by sequencing quality over time. Box plot of sequence quality per experiment over time. (a) Data distribution by total number of sequence reads per experiment. (b) Data distribution by median sequence read length per experiment. [file gix029_Supp.zip › supplementary_figure4.pdf]

a

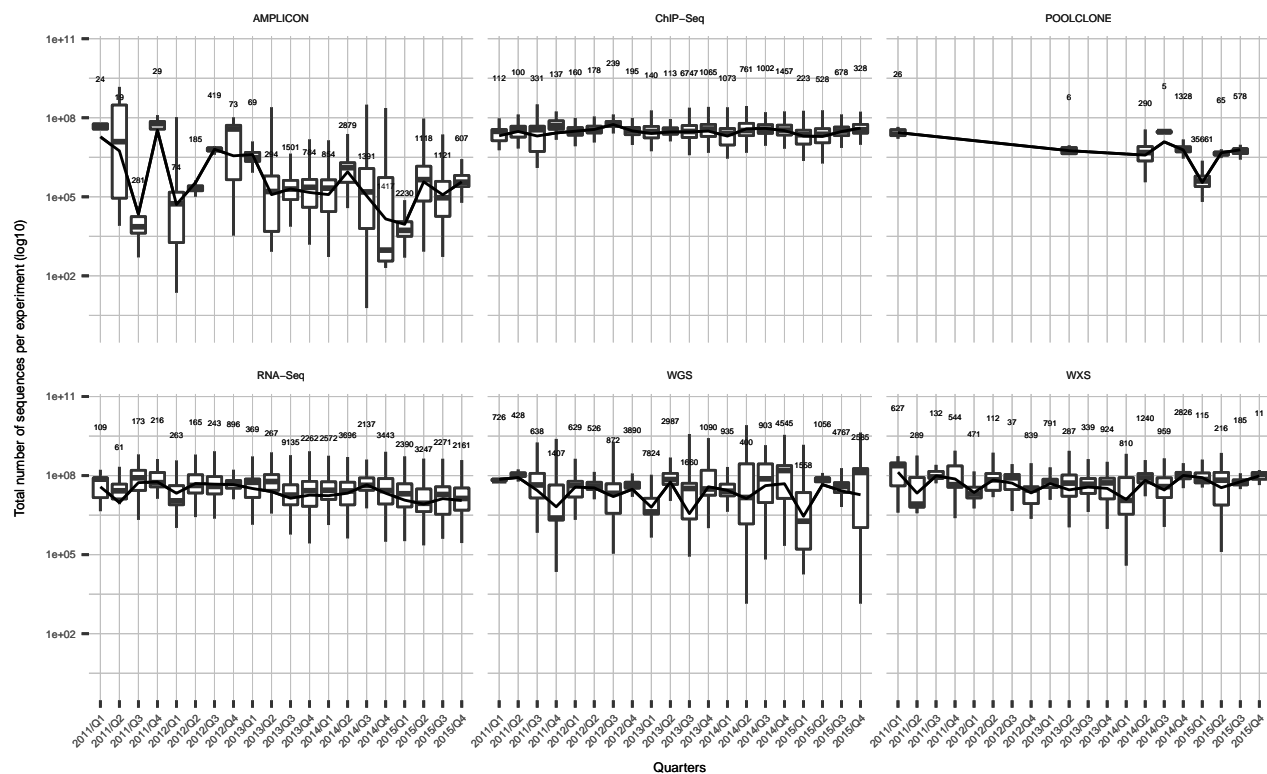

b

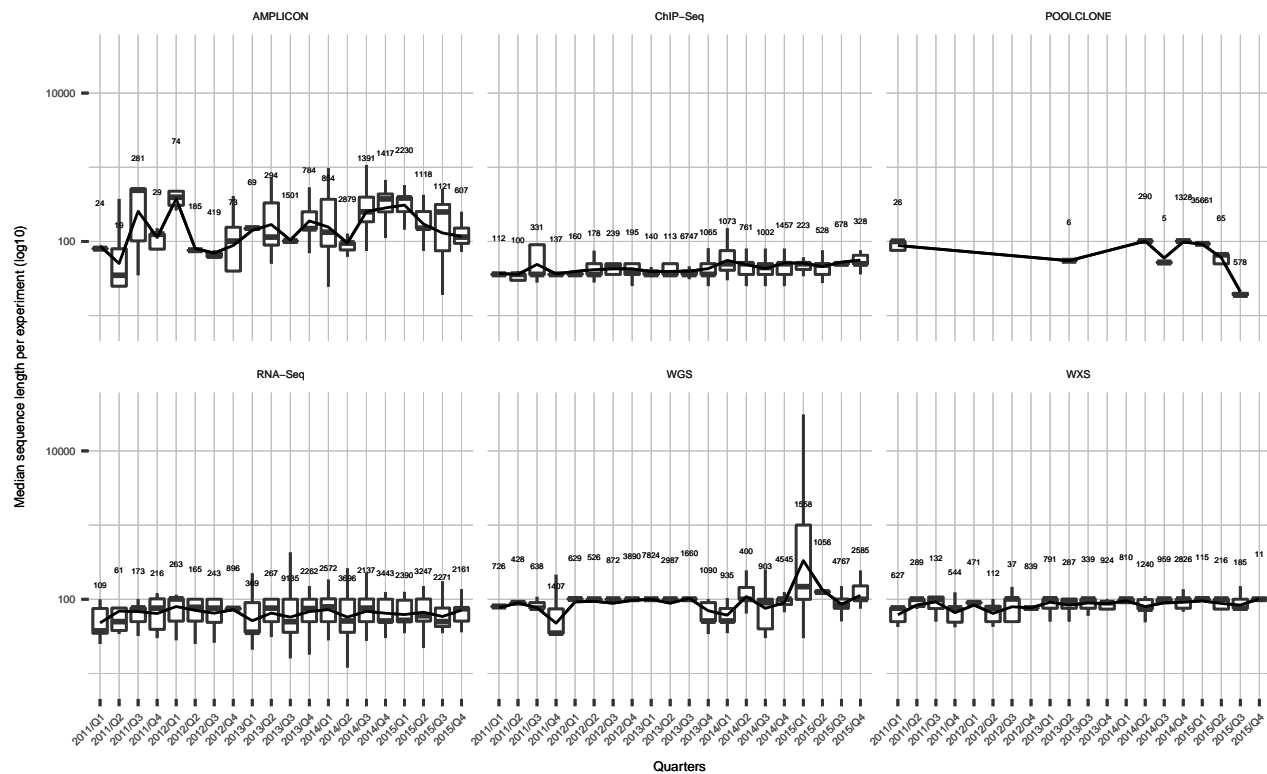

Supplement: Supplemental material — Supplementary Figure 1: Data distribution of sequencing throughput for each set of metadata. (a–e) Histograms of sequencing throughput (a), separated by library strategy (b), library source (c), top 20 taxonomic scientific names (d), and instrument manufacturer (e). Supplementary Figure 2: Data distribution of base call accuracy for each set of metadata. (a–e) Histograms of base call accuracy (a), separated by library strategy (b), library source (c), top 20 taxonomic scientific names (d), and instrument manufacturer (e). Supplementary Figure 3: Data distribution by N content. (a–f) Histograms of N content percentage per experiment. Histograms of base call failure of overall (a), separated by library strategy (b), library source (c), sample organism (d), instrument manufacturer (e), and year of data submission (f). The y-axis is log 10 scale. Supplementary Figure 4: Human data distribution for each library strategy separated by instrument manufacturer. (a–d) Histograms separated by the top 6 library strategies and instrument. Data distribution is by the total number of sequences (a), median read length (b), sequencing throughput (c), and median base call accuracy (d) per experiment. Supplementary Figure 5: Change of data distribution by sequencing quality over time. Box plot of sequence quality per experiment over time. (a) Data distribution by total number of sequence reads per experiment. (b) Data distribution by median sequence read length per experiment. [file gix029_Supp.zip › supplementary_figure5.pdf]
