# Supplementary material for: Calculating the quality of public high-throughput sequencing data to obtain a suitable subset for reanalysis from the Sequence Read Archive
Source: Gigascience. 2017 Apr 25;6(6):1–8. doi: 10.1093/gigascience/gix029 (PMC5459929; doi:10.1093/gigascience/gix029)
Supplement: Response_to_reviewer_comments_Original_Submission.pdf [file gix029_Response_to_reviewer_comments_Original_Submission.pdf]

GIGA-D-17-00014
Calculating quality of public high-throughput sequencing data to obtain suitable subset for reanalysis from the Sequence Read Archive
Tazro Ohta; Takeru Nakazato; Hidemasa Bono
GigaScience

We would like to thank the reviewers for their positive comments and constructive suggestions, which help to improve the quality of this manuscript. Our response follows (the reviewer's comments are in italics).

Response to the reviewers' comments:

Reviewer #1: It is either stated or implied several times in this manuscript that fastqc is the only way to filter between datasets. I think it's important to note that there are many other ways that one could filter large datasets (such as by sufficiency of metadata), or even assess quality (by mapping quality).

Reply: We agree with the reviewer's assessment. The text in the background section of page 4 was modified as "To select an appropriate amount of data, sequencing quality is usually used to ensure that the data are sufficient for an analysis; however, only natural language metadata described by data submitter and a few quality information such as total sequence bases are available for public sequencing data to filter the number of dataset. there are currently no detailed quality information available for public data. Categorical values described in metadata can be used to filter the data, but they are not enough to retrieve dataset in smaller resolution.".

This statement seems like an oversimplification -- This is because users prefer to collect data from a single project that had a sufficient number of samples and that were produced by experiments under reliable conditions, thus ensuring the quality of the data. -- while undoubtedly a major use case. That said, a modification would be prudent, and a reference would be fantastic, if you have one.

Reply: We could not find a proper reference to prove this use case is the major one from a neutral perspective, unfortunately. We modified the text as "This is probably because users prefer to collect data from a single project that had a sufficient number of samples and that were produced by experiments under reliable conditions, thus ensuring the quality of the data".

The other thing I would recommend is more documentation in the README of the Github repository.

Reply: Thank you for your suggestion. We have added detail usage and description of what users can do with the code in the repository. https://github.com/inutano/sra-quanto

With the exception of these minor changes, in my opinion, this is a useful tabulation of the relative quality of data in the SRA.

Reply: Thank you for your positive comments. We really appreciate it.

Reviewer #2: Overall this paper should be accepted with minor corrections. In the first instance this paper does a thorough systematic study of data deposited in the SRA and is able to draw some conclusions about the overall quality of the data deposited there. I would have liked to have the authors follow up more on their conclusions from the data but nonetheless it makes a useful contribution.

Reply: Thank you for your positive feedback.

On some relatively minor points, I think the authors should change the colour code on their plots so that the most common data has dark colours. The most common data is always yellow which makes viewing the plots very hard. The plots of counts versus Percent N content per experiment weould have been more informative if a log-scale had been used on the vertical axis.

Reply: As suggested by the reviewer, we have changed the colouring of the plot of Figure 2. To make the comparison easier, we have changed the other plots to be separated by categorical values, not be colour-coded. The percent N content plot was re-drawn using log10-scale.

Reviewer #3: Overall the manuscript describes a useful tool and could help people make better use of previously published data, which is always a good thing. There are however a number of minor issues that should be addressed;

Reply: Thank you for your positive comments.

1 - Question, are there any works previously published on the accuracy of the metadata supplied by submitters? e.g. you split the data into WGS, amplicon etc... but what proportion of those are erroneous? I believe there are a number of users that submit everything as WGS regardless of the actual strategy, if that is a significant number would it effect your findings for (a) the WGS data, or (b) the much smaller proportions of other datatypes?

Reply: Unfortunately, we could not find the suitable reference article which investigated the correctness in categorical metadata of SRA, for example, sequencing methods or sample organisms. We also asked the curators of DDBJ SRA, but they do not know such investigations regarding correctness of the metadata. However, we added a reference, which mentioned that the metadata of library construction described by data submitters are not satisfying the demand from the perspective of data reuse, to mention that there may be errors in metadata that affect the interpretation of the result.

2 - The figshare link cited is to version1, there is now a version2, but both of them appear to have either corrupt files or the files removed, I cannot download the file.
https://dx.doi.org/10.6084/m9.figshare.4498907.v1

Reply: The version 2 has been created since the curator of the figshare has asked us to change the title of the upload to more detailed one after we submitted the manuscript. The file itself was unchanged. We confirmed that the file was not able to be downloaded. The figshare support told us that it was the error of the system, so we have uploaded the same file again, and confirmed it is now downloadable. We also fixed the manuscript to cite the version 2. We are sorry for your inconvenience.

3 - An example query. Perhaps it would be useful to end users to be shown how one could use the SPARQL end-point in co-ordination with the SRA to refine their search to only return SRA runs with a specific quality criteria?
e.g. If I was interested in pulling all human metagenomic WGS reads of high quality, and above an average length of 150bp, would that be possible using the information they have in the SPARQL end-point?

Reply: We added two more example SPARQL queries on the NBDC RDF Portal. The first one is to retrieve top 50 high-throughput sequencing runs with filtering by base call quality, and the second is to calculate average sequencing throughput of the sequencing runs with more than 500 read length. We also added the text in discussion, page 13 to mention that example queries are available. For your question, unfortunately, to search sequencing data with quality and metadata is currently not possible via SPARQL endpoint. It is because that we do not have a suitable RDF model for SRA metadata, which is why we are providing tabular data as primary data for this manuscript. We are working with members of DDBJ to create SRA data model in RDF, so we hope we can provide such highly flexible data search via SPARQL soon. We also modified the text so that the linked data is only available for statistics.

4 - "We developed an ontology to describe sequencing quality information, namely, sequence statistics ontology, and also published it in the NBDC RDF portal."
This statement is perhaps over-selling the Sequence Statistic controlled vocabulary by saying its an "Ontology", I have downloaded the OWL file of it from github, and looked for it in the NBDC RDF portal and all I can find is list of terms they have used, with some hierarchical relationships, unfortunately I can find no evidence of a single definition of any of the terms. These need to be defined before anyone can understand them or reuse them (and given the authors are preaching about reuse, it seems only fair for them to make the effort on that front).

Reply: We agree with the reviewer's assessment. Now we have added the annotation of the ontology and definition of the terms. Thank you for pointing out, we are sure that this is very important act for this manuscript.

5 - I question the usefulness of some of the charts, particularly those comparing vendors, the sheer over-representation of Illumina means that all other vendors details are virtually indiscernible in the combined charts (e.g. p24 fig D), perhaps there is a better way to compare/contrast those figures?

Reply: We agree with the reviewer’s comment. We changed the figure to separate the plots by vendors or other categories.

6 - Further work; is there a plan to maintain this information either in real time or at specific time intervals moving forward so as new data being submitted to the SRA can be checked in this manner?

Reply: Yes, we are currently working on the data submitted in 2016. We will update the tabular data in figshare and RDF data in NBDC RDF Portal. The text "We will continue to calculate sequence statistics for future data submission and update the summary." has been added in the discussion section.

7 - Finally, a question to the editors (as well as the authors); would it be appropriate to give members of the INSDC (Ilene Karsch-Mizrachi, NCBI, Guy Cochrane, EBI and Toshihisa Takagi, DDBJ) a preview of the manuscript and ask for a response prior to publication? I'm thinking along the lines of giving them the opportunity to pledge support for this venture as a means to enhance their data reuse. I know from previously working with the ENA that they are always keen to do what they can to help/enable third party tools like this.

Reply: We welcome the review by INSDC members, it would be very helpful to improve the output of this manuscript. We may have to note that we have already asked Prof. Toshihisa Takagi to review this manuscript before submission, and he gave many helpful comments (mentioned in Acknowledgement), though it was not as the director of DDBJ.
