# Supplementary material for: Calculating the quality of public high-throughput sequencing data to obtain a suitable subset for reanalysis from the Sequence Read Archive
Source: Gigascience. 2017 Apr 25;6(6):1–8. doi: 10.1093/gigascience/gix029 (PMC5459929; doi:10.1093/gigascience/gix029)
Supplement: Response_to_reviewer_comments_Revision_1.pdf [file gix029_Response_to_reviewer_comments_Revision_1.pdf]

GIGA-D-17-00014
Calculating quality of public high-throughput sequencing data to obtain suitable subset for reanalysis from the Sequence Read Archive
Tazro Ohta; Takeru Nakazato; Hidemasa Bono
GigaScience

The text of data availability section is fixed and the related citations are added. We would like to thank the editors and the editorial team.
