# Supplementary material for: Calculating the quality of public high-throughput sequencing data to obtain a suitable subset for reanalysis from the Sequence Read Archive
Source: Gigascience. 2017 Apr 25;6(6):1–8. doi: 10.1093/gigascience/gix029 (PMC5459929; doi:10.1093/gigascience/gix029)
Supplement: Reviewer_2_Report_(Original_Submission).pdf [file gix029_Reviewer_2_Report_(Original_Submission).pdf]

Reviewer Report

Title: "Calculating quality of public high-throughput sequencing data to obtain suitable subset for reanalysis from the Sequence Read Archive"

Version: Original Submission **Date:** 2/5/2017

Reviewer name: Hugh Shanahan

Reviewer Comments to Author:

Overall this paper should be accepted with minor corrections. In the first instance this paper does a thorough systematic study of data deposited in the SRA and is able to draw some conclusions about the overall quality of the data deposited there. I would have liked to have the authors follow up more on their conclusions from the data but nonetheless it makes a useful contribution.

On some relatively minor points, I think the authors should change the colour code on their plots so that the most common data has dark colours. The most common data is always yellow which makes viewing the plots very hard. The plots of counts versus Percent N content per experiment weould have been more informative if a log-scale had been used on the vertical axis.

Methods

Are the methods appropriate to the aims of the study, are they well described, and are necessary controls included? Yes

Conclusions

Are the conclusions adequately supported by the data shown? Yes

Reporting Standards

Does the manuscript adhere to the journal's guidelines on minimum standards of reporting? Yes

Statistics

Are you able to assess all statistics in the manuscript, including the appropriateness of statistical tests used? There are no statistics in the manuscript.

Quality of Written English

Please indicate the quality of language in the manuscript: Acceptable

Declaration of Competing Interests

Please complete a declaration of competing interests, considering the following questions:

- Have you in the past five years received reimbursements, fees, funding, or salary from an organisation that may in any way gain or lose financially from the publication of this manuscript, either now or in the future?
- Do you hold any stocks or shares in an organisation that may in any way gain or lose financially from the publication of this manuscript, either now or in the future?
- Do you hold or are you currently applying for any patents relating to the content of the manuscript?
- Have you received reimbursements, fees, funding, or salary from an organization that holds or has applied for patents relating to the content of the manuscript?
- Do you have any other financial competing interests?
- Do you have any non-financial competing interests in relation to this paper?

If you can answer no to all of the above, write 'I declare that I have no competing interests' below. If your reply is yes to any, please give details below.

I declare that I have no competing interests.

I agree to the open peer review policy of the journal. I understand that my name will be included on my report to the authors and, if the manuscript is accepted for publication, my named report including any attachments I upload will be posted on the website along with the authors' responses. I agree for my report to be made available under an Open Access Creative Commons CC-BY license (http://creativecommons.org/licenses/by/4.0/). I understand that any comments which I do not wish to be included in my named report can be included as confidential comments to the editors, which will not be published.

I agree to the open peer review policy of the journal

To further support our reviewers, we have joined with Publons, where you can gain additional credit to further highlight your hard work (see: https://publons.com/journal/530/gigascience). On publication of this paper, your review will be automatically added to Publons, you can then choose whether or not to claim your Publons credit. I understand this statement.

Yes
