# Supplementary material for: Calculating the quality of public high-throughput sequencing data to obtain a suitable subset for reanalysis from the Sequence Read Archive
Source: Gigascience. 2017 Apr 25;6(6):1–8. doi: 10.1093/gigascience/gix029 (PMC5459929; doi:10.1093/gigascience/gix029)
Supplement: Reviewer_3_Report_(Original_Submission).pdf [file gix029_Reviewer_3_Report_(Original_Submission).pdf]

Reviewer Report

Title: "Calculating quality of public high-throughput sequencing data to obtain suitable subset for reanalysis from the Sequence Read Archive"

Version: Original Submission **Date:** 2/6/2017

Reviewer name: Christopher Hunter, Ph.D.

Reviewer Comments to Author:

Overall the manuscript describes a useful tool and could help people make better use of previously published data, which is always a good thing. There are however a number of minor issues that should be addressed;

1 - Question, are there any works previously published on the accuracy of the metadata supplied by submitters? e.g. you split the data into WGS, amplicon etc... but what proportion of those are erroneous? I believe there are a number of users that submit everything as WGS regardless of the actual strategy, if that is a significant number would it effect your findings for (a) the WGS data, or (b) the much smaller proportions of other datatypes?

2 - The figshare link cited is to version1, there is now a version2, but both of them appear to have either corrupt files or the files removed, I cannot download the file.

https://dx.doi.org/10.6084/m9.figshare.4498907.v1

3 - An example query. Perhaps it would be useful to end users to be shown how one could use the SPARQL end-point in co-ordination with the SRA to refine their search to only return SRA runs with a specific quality criteria?

e.g. If I was interested in pulling all human metagenomic WGS reads of high quality, and above an average length of 150bp, would that be possible using the information they have in the SPARQL end-point?

4 - "We developed an ontology to describe sequencing quality information, namely, sequence statistics ontology, and also published it in the NBDC RDF portal."

This statement is perhaps over-selling the Sequence Statistic controlled vocabulary by saying its an "Ontology", I have downloaded the OWL file of it from github, and looked for it in the NBDC RDF portal

and all I can find is list of terms they have used, with some hierarchical relationships, unfortunately I can find no evidence of a single definition of any of the terms. These need to be defined before anyone can understand them or reuse them (and given the authors are preaching about reuse, it seems only fair for them to make the effort on that front).

5 - I question the usefulness of some of the charts, particularly those comparing vendors, the sheer over-representation of Illumina means that all other vendors details are virtually indiscernible in the combined charts (e.g. p24 fig D), perhaps there is a better way to compare/contrast those figures?

6 - Further work; is there a plan to maintain this information either in real time or at specific time intervals moving forward so as new data being submitted to the SRA can be checked in this manner?

7 - Finally, a question to the editors (as well as the authors); would it be appropriate to give members of the INSDC (Ilene Karsch-Mizrachi, NCBI, Guy Cochrane, EBI and Toshihisa Takagi, DDBJ) a preview of the manuscript and ask for a response prior to publication? I'm thinking along the lines of giving them the opportunity to pledge support for this venture as a means to enhance their data reuse. I know from previously working with the ENA that they are always keen to do what they can to help/enable third party tools like this.

Regards

Chris

Methods

Are the methods appropriate to the aims of the study, are they well described, and are necessary controls included? Yes

Conclusions

Are the conclusions adequately supported by the data shown? Yes

Reporting Standards

Does the manuscript adhere to the journal’s guidelines on minimum standards of reporting? Yes

Statistics

Are you able to assess all statistics in the manuscript, including the appropriateness of statistical tests used? There are no statistics in the manuscript.

Quality of Written English

Please indicate the quality of language in the manuscript: Acceptable

Declaration of Competing Interests

Please complete a declaration of competing interests, considering the following questions:

- Have you in the past five years received reimbursements, fees, funding, or salary from an organisation that may in any way gain or lose financially from the publication of this manuscript, either now or in the future?
- Do you hold any stocks or shares in an organisation that may in any way gain or lose financially from the publication of this manuscript, either now or in the future?
- Do you hold or are you currently applying for any patents relating to the content of the manuscript?
- Have you received reimbursements, fees, funding, or salary from an organization that holds or has applied for patents relating to the content of the manuscript?
- Do you have any other financial competing interests?
- Do you have any non-financial competing interests in relation to this paper?

If you can answer no to all of the above, write 'I declare that I have no competing interests' below. If your reply is yes to any, please give details below.

I am a current employee of GigaDB, which is a subsidiary of GigaScience Journal.

I agree to the open peer review policy of the journal. I understand that my name will be included on my report to the authors and, if the manuscript is accepted for publication, my named report including any attachments I upload will be posted on the website along with the authors' responses. I agree for my report to be made available under an Open Access Creative Commons CC-BY license (http://creativecommons.org/licenses/by/4.0/). I understand that any comments which I do not wish to be included in my named report can be included as confidential comments to the editors, which will not be published.

I agree to the open peer review policy of the journal

To further support our reviewers, we have joined with Publons, where you can gain additional credit to further highlight your hard work (see: https://publons.com/journal/530/gigascience). On publication of this paper, your review will be automatically added to Publons, you can then choose whether or not to claim your Publons credit. I understand this statement.

Yes
